# Antifungal potential of secondary metabolites involved in the interaction between citrus pathogens

**DOI:** 10.1038/s41598-019-55204-9

**Published:** 2019-12-09

**Authors:** Jonas Henrique Costa, Cristiane Izumi Wassano, Célio Fernando Figueiredo Angolini, Kirstin Scherlach, Christian Hertweck, Taícia Pacheco Fill

**Affiliations:** 1 Institute of Chemistry, University of Campinas, CP 6154, 13083-970 Campinas, SP Brazil; 2 Center for Natural and Human Sciences, Federal University of ABC, 09210-580 Santo André, SP Brazil; 3 Department of Biomolecular Chemistry, Leibniz Institute for Natural Product Research and Infection Biology – Hans Knöll Institute, Jena, Germany; 4 Chair of Natural Product Chemistry, Friedrich Schiller University Jena, 07743 Jena, Germany

**Keywords:** Antifungal agents, Fungi

## Abstract

Numerous postharvest diseases have been reported that cause substantial losses of citrus fruits worldwide. *Penicillium digitatum* is responsible for up to 90% of production losses, and represent a problem for worldwide economy. In order to control phytopathogens, chemical fungicides have been extensively used. Yet, the use of some artificial fungicides cause concerns about environmental risks and fungal resistance. Therefore, studies focusing on new approaches, such as the use of natural products, are getting attention. Co-culture strategy can be applied to discover new bioactive compounds and to understand microbial ecology. Mass Spectrometry Imaging (MSI) was used to screen for potential antifungal metabolites involved in the interaction between *Penicillium digitatum* and *Penicillium citrinum*. MSI revealed a chemical warfare between the fungi: two tetrapeptides, deoxycitrinadin A, citrinadin A, chrysogenamide A and tryptoquialanines are produced in the fungi confrontation zone. Antimicrobial assays confirmed the antifungal activity of the investigated metabolites. Also, tryptoquialanines inhibited sporulation of *P. citrinum*. The fungal metabolites reported here were never described as antimicrobials until this date, demonstrating that co-cultures involving phytopathogens that compete for the same host is a positive strategy to discover new antifungal agents. However, the use of these natural products on the environment, as a safer strategy, needs further investigation. This paper aimed to contribute to the protection of agriculture, considering health and ecological risks.

## Introduction

Citrus fruits have an important impact on world’s economy since they have the largest production compared with other fruits^1^; for example, the global orange production for 2018/19 is forecast to reach 54.3 million tons^2^. The major factor affecting the quality of citrus is the postharvest fungal diseases, in particular the green mold caused by *Penicillium digitatum* that is responsible for 90% of citrus losses during postharvest period^1,3^.

To control post-harvest diseases, synthetic fungicides are widely used^4^, causing health and environmental issues^5^. Furthermore, some fungi strains have developed resistance to commonly used fungicides^6^. As antifungal resistance is becoming a significant concern, the search for new bioactive compounds has an important role to bypass the extensive use of fungicides^7^.

Microorganisms are one of the main sources for natural products with useful biological activities, i.e., potential antifungals, antibiotics, anticancer agents, surfactants^8,9^. However, besides the genetic potential and diversity of microbes, many microbial biosynthetic genes are not activated in the unnatural cultivation conditions used in laboratory and only a few part of the metabolites are accessible^10^. Several strategies are implemented to overcome the limitations in natural products discovery from microbial sources, i.e., OSMAC approach^10,11^, the use of epigenetic modifications^12,13^ and co-cultivation^14^.

Co-cultures that mimic natural environments, exhibiting microbial competition for limited space and nutrients, have revealed to be a major ecological force that could activate silent gene clusters and defense mechanisms that can lead to the production of bioactive secondary metabolites^14–17^. Co-culture experiments are highly relevant for allowing not only the identification of new compounds, but also to investigate chemical events that govern interactions between microorganisms in nature^18–21^.

To date, there is no information about how citrus pathogen fungi interact with other microorganisms in the same environment and what are the mechanisms of attack and defense against endophytic microorganisms or other phytopathogens. This information may lead to the discovery of new secondary metabolites involved in microbial interactions and provide better knowledge about the importance of microbial ecology during infection process.

Here we show an interaction between *Penicillium digitatum* and *Penicillium citrinum*, aiming to search for new antifungal compounds that could be used to control postharvest diseases. Considering this purpose, co-culture strategy and MSI were applied to induce the production of secondary metabolites and provide initial insights concerning their biological role based on their spatial distribution in the interaction. The metabolites of interest were isolated and all the structures were elucidated based on Mass spectrometry and NMR experiments. Yet, antifungal assays and confocal microscopy analyses were performed in order to investigate the potential of the fungal metabolites as new antifungal agents.

## Materials and Methods

### Fungi culture

The *P. digitatum* (PD) strain used in the studies is deposited with the Spanish Type Culture Collection (CECT) under the accession code CECT20796. *P. citrinum* (PC) strain was provided by Sylvio Moreira Citrus Center (Cordeirópolis, SP, Brazil). *P. digitatum* and *P. citrinum* were cultivated on commercial potato dextrose agar (PDA) (Acumedia). PDA was autoclaved at 103 KPa (121 °C) for 15 min. PDA plates were stored at 25 °C for 7 days in darkness. Spores were harvested by washing the agar surface with sterile distilled water and diluted to a final concentration of 10^6^ or 10^5^ spore mL^−1^.

### Co-culture growth conditions

*In vitro* co-culture was prepared in 20 mL of PDA plates and 5 µL of each fungal spore solution (10^6^ spore mL^−1^) was inoculated, on opposite sides. The plates were incubated in darkness at 25 °C for 7 days.

For MSI analyses and confocal microscopy, the *in vitro* co-culture was prepared by placing a sterile microscope slide in the Petri dish, followed by the pouring of 11 ml of PDA^22^ and the inoculums were made above the microscope slide. The plates were incubated in darkness at 25 °C for 72 h.

For *in vivo* co-culture, mature oranges (*Citrus sinensis*) were surface sterilized and wounded^23^. A small piece of PDA containing *P. citrinum* was inoculated in the wound site. Infected and control oranges were stored in sterile 500 mL beakers, in darkness at 25 °C. After 10 days, the fruits were wounded on the opposite equatorial region of *P. citrinum* inoculum and infected with *P. digitatum* 10^6^ spore mL^−1^ solution. The fruits were stored for more 5 days in darkness at 25 °C.

### Mass Spectrometry Imaging (MSI) analysis and MS image generation

After the incubation period, the microscope slides were removed from the Petri dishes and put in a vacuum desiccator, for 1 hour, for complete agar dehydration (Angolini *et al*., 2015). MSI analyses were performed directly on the microscope containing the co-culture, in positive mode, using a desorption electrospray ionization (DESI) source Prosolia Model Omni Spray 2D^®^-3201) coupled to a Thermo Scientific QExactive^®^ Hybrid Quadrupole-Orbitrap Mass Spectrometer. MSI data was acquired with a mass resolving power of 70.000 at *m/z* 200. The DESI configuration used was the same set by previous work^22^. Images were generated with a bin width of Δ*m*/*z* =  ± 0.07 using Firefly data conversion software (version 2.1.05) and processed using BioMap software (version 3.8.0.4) developed by Novartis Institutes for BioMedical Research. In BioMap, color scaling was adjusted to a fixed value during the processing of each image. MS spectra were processed with Xcalibur software (version 3.0.63) developed by Thermo Fisher Scientific.

### Extraction of metabolites from the co-culture experiments

The whole contents of the co-culture *in vitro* were cut into small pieces and transferred to an Erlenmeyer flask. The extraction was performed using methanol. The flasks were sonicated during 1 h in ultrasonic bath and vacuum filtered. Solvent was removed under reduced pressure and the final extract stored at −20 °C.

For *in vivo* co-culture, the orange peels were cut (2 cm × 2 cm) in the interface zone between the microorganisms and extraction was performed with 5 mL of methanol during 1 h in ultrasonic bath. Extracts were filtered, dried under N_2_ and stored at −20 °C.

### Mass Spectrometry analysis (MS)

Extracts were diluted in methanol and analyzed on a Thermo Scientific QExactive^®^ Hybrid Quadrupole-Orbitrap Mass Spectrometer. Analyses were performed in the positive mode with *m/z* range of 115–1500, capillary voltage of 3.4 kV, inlet capillary temperature of 280 °C, S-lens 100 V. 5 µL of sample were injected. Stationary phase: Thermo Scientific column Accucore C18 2.6 µm (2.1 mm × 100 mm). Mobile phase: 0.1% formic acid (A) and acetonitrile (B). Eluent profile (A/B): 95/5 up to 2/98 within 15 min, hold for 5 min, up to 95/5 within 1.2 min and hold for 7.8 min. The total run time was 29 min for each run and the flow rate, 0.2 mL min^−1^. Injection volume: 5 µL.

MS/MS was performed by the collision induced dissociation (CID) with *m/z* range of 100–800 and the collision energy ranged from 10 to 50 V. The samples were directly infused by electrospray with 5.0 µL min^−1^ flow rate. MS and MS/MS data was processed with Xcalibur software (version 3.0.63) developed by Thermo Fisher Scientific.

### Metabolite separation (HPLC analysis)

Secondary metabolites separations were achieved using a Phenomenex column Luna 5 µm Phenyl-Hexyl (250 × 4.6 mm) and a SHIMADZU prominence HPLC LC-20AT, equipped with CBM-20A communication bus module, SPD-M20A photodiode array detector and SIL-20A auto sampler. Mobile phase: water (0.1% formic acid) (A) and acetonitrile (B). Eluent profile (A/B): 65/35 up to 50/50 within 50 min, up to 40/60 within 20 min. The total run time was 70 min and the flow rate of 1.0 mL min^−1^. Injection volume was 5 µL. Preparative HPLC purifications were performed on a Phenomenex column Luna 5 µm Phenyl-Hexyl (250 × 10 mm) using a Waters 1525 Binary HPLC Pump equipped with Waters 2998 Photodiode Array Detector and Waters Fraction Collector III using the same optimized gradient conditions. The flow rate was set at 4.7 mL min^−1^ and the injection volume was 200 µL.

### NMR Spectroscopy

^1^H NMR, ^13^C NMR and 2D experiments were performed on a Bruker Avance III 500 (^1^H 500.13 MHz and ^13^C 125.7 MHz) and Bruker Avance III 600 (^1^H 600.17 MHz). Deuterated chloroform (CDCl_3_; 7.23 ppm), dimethyl sulfoxide (DMSO; 2.50 ppm and 39.51 ppm) and tetramethylsilane (TMS; 0.0 ppm) were used as a solvent and internal reference. Chemical shifts (δ) were expressed in (ppm) and the coupling constants (*J*) in Hertz (Hz).

### Molecular Networking analysis

A molecular network for *P. citrinum* metabolites was created using the online workflow at Global Natural Products Social Molecular Networking (GNPS) (http://gnps.ucsd.edu). The data was filtered by removing all MS/MS peaks within + /−17 Da of the precursor *m/z*. MS/MS spectra were window filtered by choosing only the top 6 peaks in the + /−50 Da window throughout the spectrum. The data was then clustered with MS-Cluster with a parent mass tolerance of 0.2 Da and a MS/MS fragment ion tolerance of 0.2 Da to create consensus spectra. Further, consensus spectra that contained less than 2 spectra were discarded. A network was then created where edges were filtered to have a cosine score above 0.65 and more than 2 matched peaks. Further edges between two nodes were kept in the network only if each of the nodes appeared in each other’s respective top 10 most similar nodes. The spectra in the network were then searched against GNPS’ spectral libraries. The library spectra were filtered in the same manner as the input data. All matches kept between network spectra and library spectra were required to have a score above 0.5 and at least 5 matched peaks. The resulting molecular networking is available at https://gnps.ucsd.edu/ProteoSAFe/status.jsp?task=c6f716c6fd044ba985eacd96935ee0c3.

### Antifungal assays

A stock solution of the co-culture extract was prepared in methanol and further diluted in PDA to the concentration of 0.5 mg mL^−1^. 15 mL of the resultant solution was poured in a Petri dish followed by the inoculation of 15 μl of a 10^5^ spore mL^−1^ *P. digitatum* solution on the center of the agar plate. A control assay was also performed. The plates were incubated in darkness at 25 °C for 96 h.

For antifungal assays, 2.5 mL of PDA was supplemented with **6**, **9** and **10** (400 µg mL^−1^) and each solution were poured in a 6-well microplate. 5 μl of a 10^5^ spore mL^−1^ *P. digitatum* solution was inoculated on the center of each agar plate. Negative controls were performed in triplicate. The microplate was incubated in darkness at 25 °C for 96 h.

For determination of minimum inhibitory concentration (MIC) of compounds **1** and **4**, microbroth dilution assay was performed as recommended by Clinical and Laboratory Standards Institute (2008)^24^ with few modifications. Stock solutions of **1** and **4**, were prepared in water (5% methanol) and further diluted in YES media in a range of concentrations to 600 µg mL^−1^ to 1 µg mL^−1^. 195 μl of each solution were transferred to a 96-well microplate followed by the inoculation of 5 μl of a 10^5^ spore mL^−1^ *P. digitatum* solution. Assays were made in duplicate and controls in triplicate. Itraconazole (100 µg mL^−1^) were used as positive control. Negative controls were performed with methanol in YES. The microplates were incubated in darkness at 25 °C for 96 h.

### Confocal laser scanning microscopy

After the incubation period, the microscopes slides were removed from the Petri dish. The *in vitro* co-culture samples were stained with Congo Red (0.25% w/v in water) for 20 minutes and briefly washed in distilled water. Samples were analyzed with Leica TCS SP5 microscope. Excitation was by the 543 nm emission line of the He-Ne laser, and light emitted between 570 and 680 nm was collected^25^.

## Results and Discussion

### MSI reveals potential antifungals in the interaction between *P. digitatum* and *P. citrinum*

To screen for new antifungal compounds with potential to protect citrus fruits and control postharvest diseases, we applied a co-culture strategy involving *P. digitatum* and another citrus pathogen, *P. citrinum*. Co-culture is a strategy inspired by nature in which the competition between the microorganisms can induce the production of new metabolites^8^. In previous work, the co-cultivation between *Trichophyton rubrum* and *Bionectria ochroleuca* induced the production of a new sulfated analogue of PS-990, suggesting that this compound is further sulfated during the fungal interaction^26^. Also, another example of a compound derived from fungi interaction is the tetrapeptide cyclo-(_L_-leucyl-*trans*-4-hydroxy-_L_-prolyl-_D_-leucyl-*trans*-4-hydroxy-_L_-proline) isolated from the co-culture broth of *Phomopsis* sp. K38 and *Alternaria* sp. E33; the cyclic tetrapeptide exhibited moderate to high inhibitory activity against phytopathogenic fungi when compared to the commercial fungicide triadimefon^27^. Thus, co-cultivation experiments are a viable approach to find compounds that can inhibit the main citrus phytopathogens, specially, the green mold caused by *P. digitatum*.

In co-cultivation performed in both orange (*in vivo*) and synthetic media (*in vitro*) we visually observed a long-distance growth inhibition between *P. citrinum* and *P. digitatum*. In a fungi interaction, silent genes can be activated and harmful metabolites can be diffused from one partner to the other^16,28^. These induced metabolites are usually localized at the zone of confrontation in solid media of co-cultures^16^. However, regular approaches used to detect and elucidate metabolites such as mass spectrometry coupled to liquid (LC-MS) or gas (GC-MS) chromatography do not provide information about the spatial distribution of the molecules^29^.

The information about molecular spatial distribution can be obtained by mass spectrometry imaging (MSI), a powerful tool that generates images for each ion detected in the mass spectrum^16^. The use of MSI to understand microbial systems and their secondary metabolites is not new and studies where this technique was successfully applied can be found in the literature^30^. By example, MSI was applied to investigate the interaction between *Bacillus subtilis* 3610 and *Streptomyces coelicolor* A3. DESI imaging of the bacterial co-culture revealed 57 signals spatially localized to bacterial colonies, leading to the identification of some secondary metabolites such as surfactin and plipastatin^31^. Furthermore, MSI analysis also showed that *S. coelicolor* has the production of certain secondary metabolites inhibited in the presence of *B. subtilis*, revealing an interaction between bacteria^31^.

**Figure 1:**
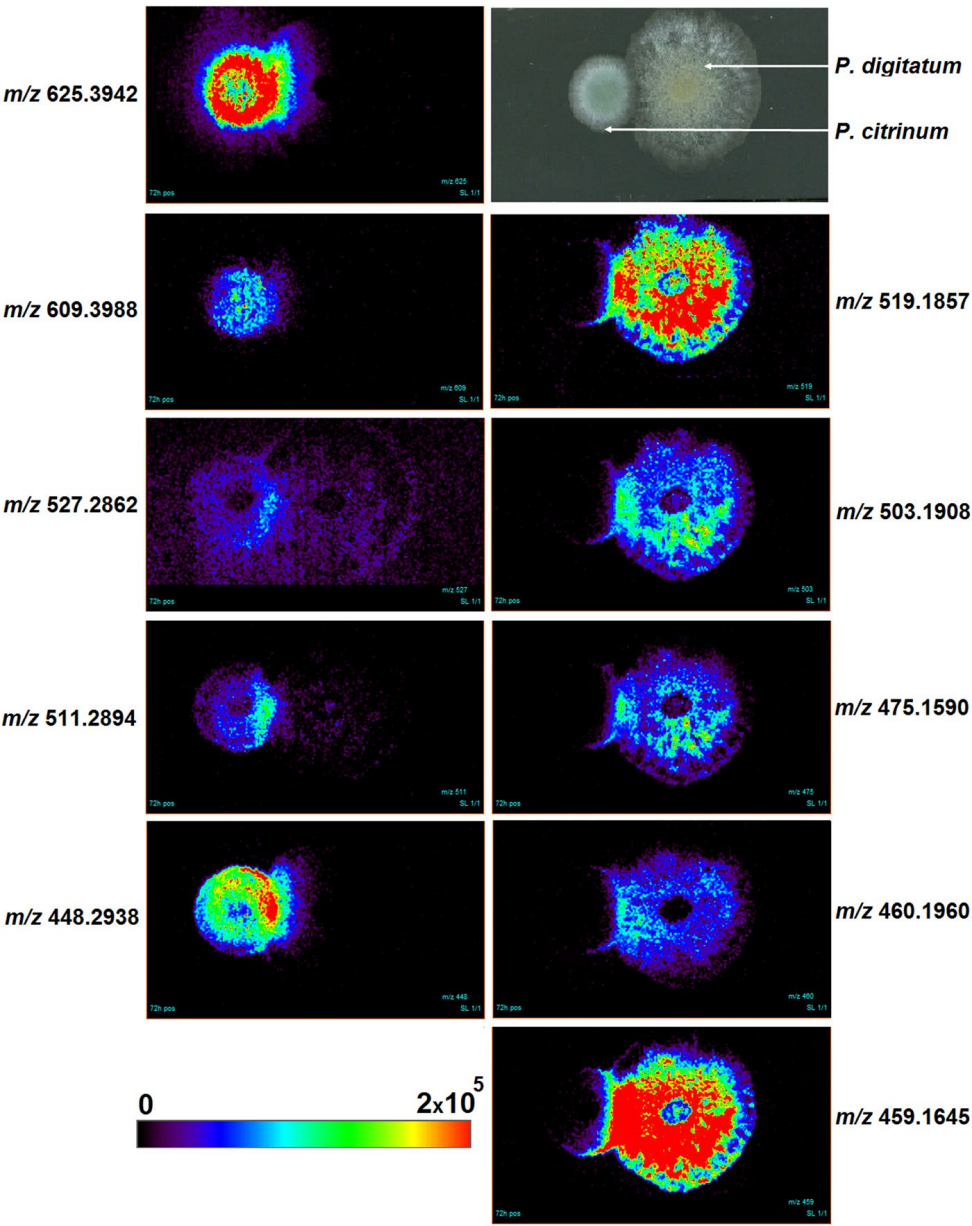
Imaging analysis by (+) DESI-MS of citrus pathogens co-culture. Each MS image shows the spatial distribution of a *m/z* ratio over the co-culture surface. All images are plotted on the same color scale from 0 (black) to 2 × 10^5^ (red) yet ion concentration cannot be compared across images since differences in chemical structures can lead to variation in ionization efficiency^30^.

**Figure 2:**
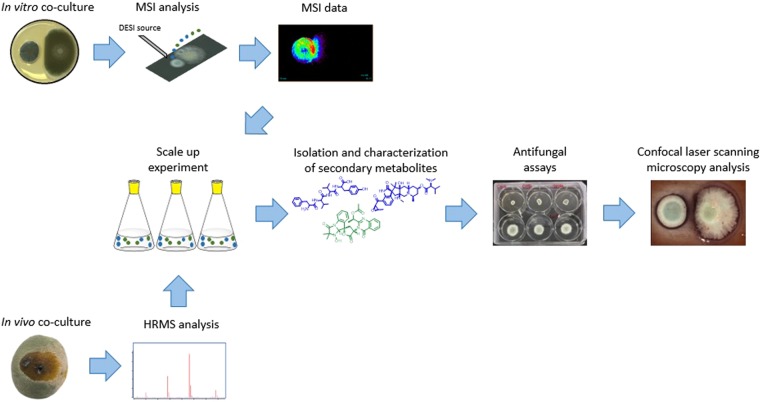
Overview of the experimental setup and strategies applied to analyze the secondary metabolites involved in citrus pathogens interaction. *P. digitatum* and *P. citrinum* were co-cultured in orange (*in vivo*) and in PDA media (*in vitro*) to induce the production of secondary metabolites. MSI was applied, *in vitro*, to detect the secondary metabolites produced in the zone of confrontation. HRMS analysis was performed to confirm the fungus-fungus interaction *in vivo*. The metabolites of interest were isolated from a scale up experiment. Subsequently, antifungal assays and confocal laser scanning microscopy analysis were performed to investigate antimicrobial activity and fungal cell morphology, respectively.

Therefore, to detect the secondary metabolites involved in the interaction between the citrus pathogens, we applied DESI-MSI directly on the surface of the co-culture agar, to visualize the diffusion of compounds to the zone of confrontation. MSI signals were obtained for ions [M + H]^+^ at *m/z* 519.1857, *m/z* 503.1908, *m/z* 475.1590, *m/z* 460.1960, *m/z* 459.1645, *m/z* 625.3942, *m/z* 609.3988, *m/z* 527.2862, *m/z* 511.2894 and *m/z* 448.2938 (Figs. S1–S10). We observed that all the ions mentioned were detected and concentrated in the zone of confrontation between the fungi (Fig. 1). These compounds may be related to the fungus-fungus interaction and could be potentially new antimicrobial agents. Ions at *m/z* 625, 609, 527, 511 and 448 were produced by *P. citrinum*, while ions at *m/z* 519, 503, 475, 460 and 459, seemed to be a counter-attack of *P. digitatum*, revealing a chemical warfare between these two citrus pathogens. The ions detected *in vitro* through MSI analyses were also detected in the extracts of the co-cultures *in vivo* (Figs. S11–S20) using oranges as substrate (Fig. 2).

To characterize the metabolites involved in the interaction, a co-culture extract was obtained from a scale up cultivation experiment and the compounds of interest, detected initially through MSI analyses, were isolated by preparative HPLC and characterized through tandem mass spectrometry and NMR analyses.

**Figure 3:**
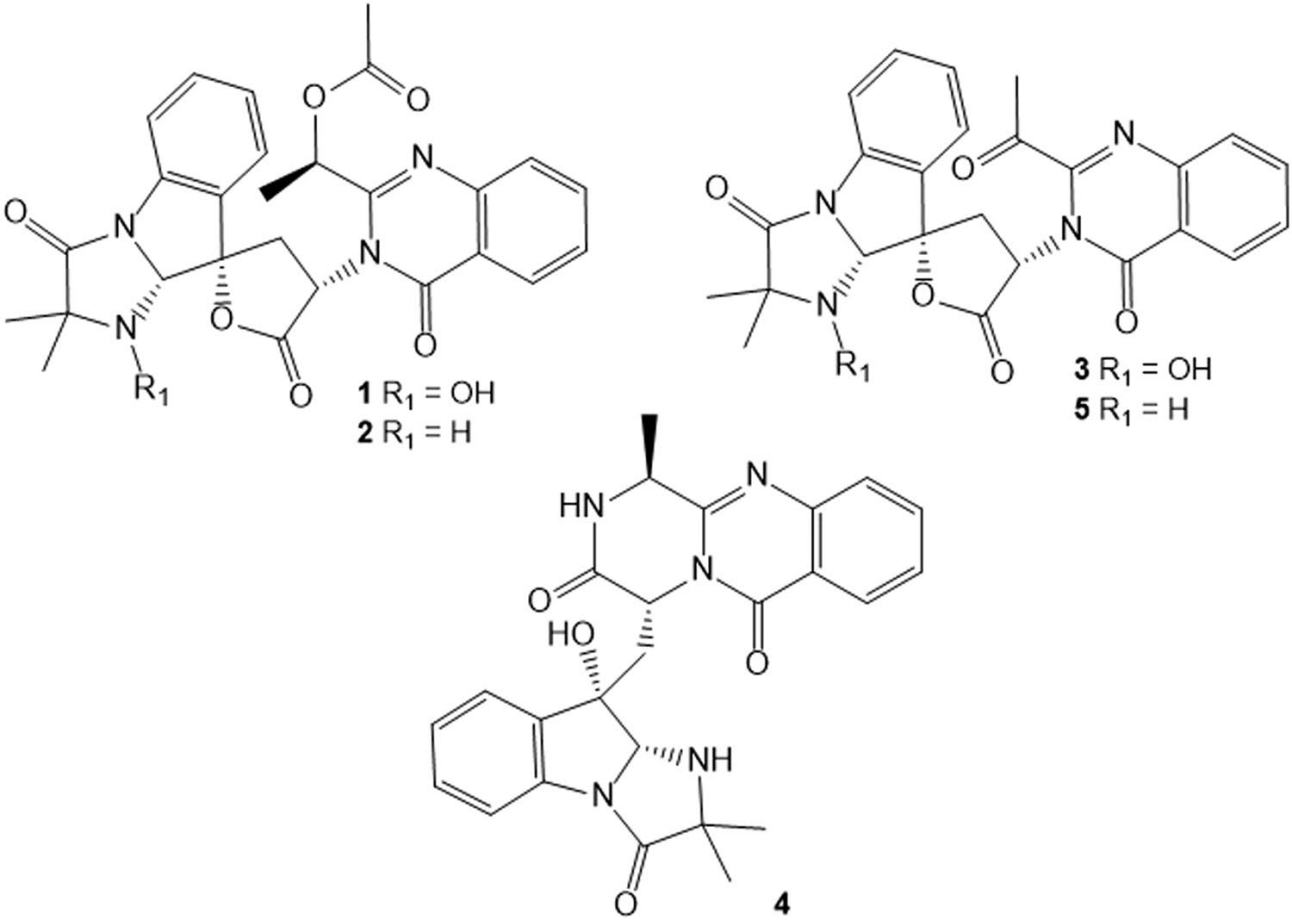
Chemical structures of indole alkaloids produced by *P. digitatum*: tryptoquialanine A (**1**), tryptoquialanine C (**2**), tryptoquialanone (**3**), 15-dimethyl-2-epi-fumiquinazoline A (**4**) and deoxytryptoquialanone (**5**). These compounds were detected through DESI-MSI in the fungal confrontation zone.

Through the exact masses obtained by DESI-MSI analyses (Table 1) it was possible to confirm the presence of indole alkaloids produced by *P. digitatum*. Ions [M + H]^+^ at *m/z* 519.1857, *m/z* 503.1908, *m/z* 475.1590, *m/z* 460.1960 and *m/z* 459.1645 which correspond respectively to tryptoquialanine A (**1**), tryptoquialanine C (**2**), tryptoquialanone (**3**), 15-dimethyl-2-epi-fumiquinazoline A (**4**) and deoxytryptoquialanone (**5**) (chemical structures represented in Fig. 3). These compounds are part of the tryptoquialanines biosynthetic pathway^32^ and were previously detected during DESI-MSI analysis of oranges infected with the green mold disease^23^.

**Table 1:** DESI-MSI data obtained for the secondary metabolites involved in *P. digitatum* and *P. citrinum* interaction.

Compound	Ion formula ([M + H]^+^)	Calculated m/z	Experimental m/z	Error (ppm)
Tryptoquialanine A	C_27_H_27_N_4_O_7_	519.1874	519.1857	−3.4
Tryptoquialanine C	C_27_H_27_N_4_O_6_	503.1925	503.1908	−3.5
Tryptoquialanone	C_25_H_23_N_4_O_6_	475.1612	475.1590	−4.6
15-dimethyl-2-epi-fumiquinazoline A	C_25_H_26_N_5_O_4_	460.1979	460.1960	−4.2
deoxytryptoquialanone	C_25_H_23_N_4_O_5_	459.1663	459.1645	−4.0
Citrinadin A	C_35_H_53_N_4_O_6_	625.3960	625.3942	−2.8
Deoxycitrinadin A	C_35_H_53_N_4_O_5_	609.4010	609.3988	−3.7
Phe-Val-Val-Tyr	C_28_H_39_N_4_O_6_	527.2864	527.2862	−0.4
Phe-Val-Val-Phe	C_28_H_39_N_4_O_5_	511.2915	511.2894	−4.1
Chrysogenamide A	C_28_H_38_N_3_O_2_	448.2959	448.2938	−4.6

Compound **1** was reported as the major secondary metabolite produced by *P. digitatum*^33^. Yet, the deletion of *tqaA* gene, responsible to regulate **1** biosynthesis, showed that **1** is not involved in the pathogenicity of *P. digitatum* against citrus since the infection ability of the mutants was not altered^34^. The exact biological role of the tryptoquialanines is still unknown^34^ and this study provides a new biological activity of the tryptoquialanines with the involvement of these alkaloids in the fungal-fungal interaction.

The MS/MS of the ion [M + H]^+^ at *m/z* 625.3951 yielded to fragments at *m/z* 594.3533, 576.3427, 449.2430 and 431.2325 (Fig. S21), the same fragmentation pattern obtained for citrinadin A (**6**) in a study involving co-culture between *P. citrinum* and *Pseudoalteromonas sp*. OT59^19^. Also GNPS database suggested that the ion at *m/z* 625 could be citrinadin A. The tandem mass spectrum obtained for this compound shared five fragments in common with the tandem mass spectrum of **6** deposited in the database (Fig. S22). In addition, it shares similar MS/MS fragmentation pattern with citrinadin A reported by Moree *et al*. (2014). Citrinadins were reported being higher produced in co-culture situations and concentrated in co-culture interfaces, suggesting a defensive response of *P. citrinum* against other microorganisms^19,35^.

Molecular networking analysis of the isolated compounds revealed that another citrinadin is involved in the co-culture interaction. We observed that ion at *m/z* 609, detected initially by MSI analysis, is grouped in the same cluster of compound **6** (*m/z* 625) (Fig. S23), suggesting that this compound is a citrinadin-like metabolite. In a molecular networking analysis, related metabolites are grouped in same clusters since they have similar MS/MS spectrum^36^.

The exact mass obtained for ion [M + H]^+^ at *m/z* 609.4010 corresponds to a compound with molecular formula C_35_H_53_N_4_O_5_. Fragmentation pattern yielded to fragments at *m/z* 578.3583, 464.2905, 451.2586 and 433.2482 (Supplementary Fig. S24). ^1^H NMR and ^1^H-^1^H COSY spectra obtained for the purified metabolite (Figs. S25–S26 and Table S1) exhibited similar signals of a synthesized deoxycitrinadin A that was reported by Bian *et al*. (2013)^37^. The epoxide characteristic signal at δ_H_ 4.0 was absent in this derivative and a signal for vynilic hydrogen could be observed at δ_H_ 6.9, indicating the lack of the epoxide group and the presence of a double bond in comparison with citrinadin A^37^. It is the first time that deoxycitrinadin A (**7**) is reported in the literature as a secondary metabolite produced by a microorganism.

Exact masses obtained by DESI-MSI for ions [M + H]^+^ at *m/z* 527.2862 and 511.2894 indicated compounds with elemental composition of C_28_H_38_N_4_O_6_ and C_28_H_38_N_4_O_5_, respectively. MS/MS revealed that the ion at *m/z* 511 has similar structure compared to *m/z* 527, except by an absence of an oxygen atom. Fragmentation of the ion at *m/z* 527 yielded to fragments *m/z* 281, 247, 219, 182 and 120 (Fig. S27), while ion at *m/z* 511 yielded to *m/z* 265, 247, 219, 166 and 120 (Fig. S30). Same fragmentation pattern was reported for the sequence of tetrapeptides Phe-Val-Val-Tyr (**8**) of *Penicillium canescens*^38^. Yet, the tetrapeptides **8** and Phe-Val-Val-Phe (**9**) were recently reported as secondary metabolites produced by *Penicillium roqueforti*^39^. ^1^H and ^13^C NMR analyses of the isolated compounds (Figs. S28–S32 and Tables S2-S3) confirmed the results obtained through MS/MS, concluding that ions at *m/z* 527 and *m/z* 511 correspond to **8** and **9**, respectively. The production of these tetrapeptides in fungal chemical warfare is not surprising because small peptides are known for their antimicrobial activity^40^. This is the first report of **8** and **9** as secondary metabolites of *P. citrinum*.

**Figure 4:**
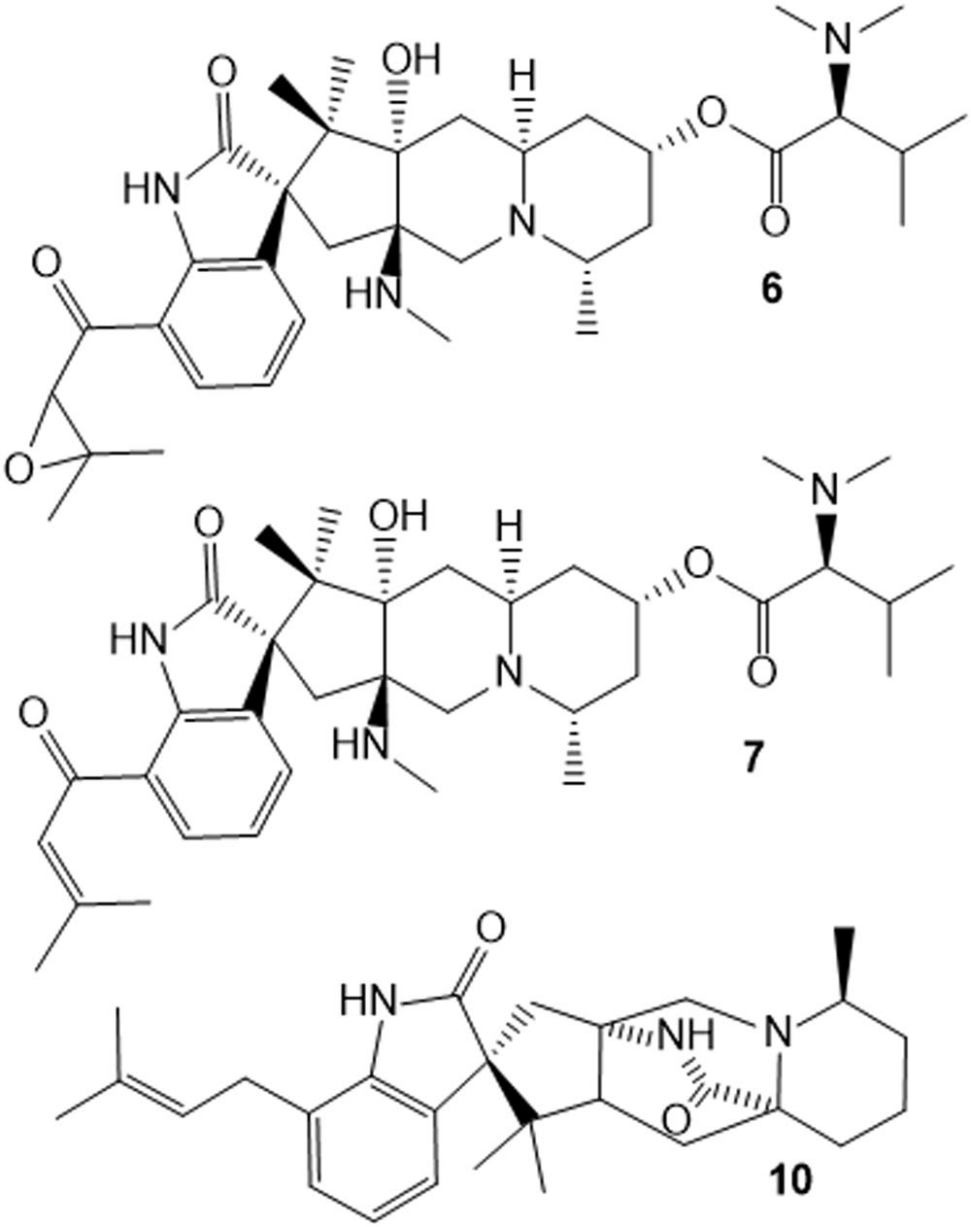
Chemical structures of secondary metabolites produced by *P. citrinum* during chemical warfare against *P. digitatum*: citrinadin A (**6**), deoxycitrinadin A (**7**) and chrysogenamide A (**10**).

For ion [M + H]^+^ at *m/z* 448.2938, the exact mass suggested a compound with molecular formula C_28_H_38_N_3_O_2_, the same composition of the secondary metabolite chrysogenamide A (**10**) (error = −4.6 ppm). Compound **10** was isolated and ^1^H and ^13^C NMR analyses (Figs. S33–S34 and Table S4) confirmed its structure; it is the first report that chrysogenamide A is involved in a fungal-fungal interaction. Compound **10** was first reported as a secondary metabolite of *Penicillium chrysogenum* No. 005, an endophytic fungus associated with the plant *Cistanche deserticola*^41^. Also, **10** was reported as a secondary metabolite of a *P. citrinum* strain^42^. The structures of the metabolites produced by *P. citrinum* are represented in Fig. 4.

### Antifungal assays

**Figure 5:**
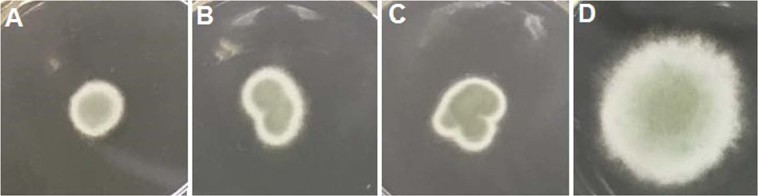
*P. digitatum* growing on PDA with 400 µg ml^−1^ of (**A**) chrysogenamide A (**B**) citrinadin A and (**C**) tetrapeptide Phe-Val-Val-Phe. In 96 h, an inhibition in radial growth is observed in the presence of the isolated metabolites when compared to (**D**) control.

We investigated the antifungal activity of the metabolites produced in the co-culture and *P. digitatum* was inoculated in agar media containing co-culture extract. Compared to the control, we observed a reduction of 67% in *P. digitatum* radial growth (Fig. S35), indicating that the metabolites involved in the fungal warfare have potential as antifungal agents. To confirm this hypothesis, we tested the isolated compounds and observed that compounds **6**, **9** and **10** (400 µg mL^−1^) reduced 48%, 41% and 61% of *P. digitatum* radial growth, respectively, when compared to control (Fig. 5), confirming the antifungal activity.

Citrinadins were found to be involved in the response of *P. citrinum* against other microorganisms in co-cultures, but the biological role of them in biological environments are still unknown to this date^19^. Compound **6** was tested for Anti-buruli ulcer activity on *Mycobacterium ulcerans* MN209, but no interesting MIC was observed^43^; also, cytotoxicity activity of **6** against leukemia and carcinoma cells was reported^44^. Our results are the first report of an antimicrobial activity of citrinadins in literature and can provide first insights about the biological role of these compounds in fungal-fungal interactions.

In addition, chrysogenamide A was never reported as an antimicrobial agent until now. Compound **10** exhibited a protective effect on neurocytes against oxidative stress-induced cell death^41^, however no other biological activity for **10** was reported in the literature.

Antifungal assays applying _D_-Phe-_L_-Val-_D_-Val-_L_-Tyr revealed that this tetrapeptide has inhibitory activity against *B. subitllis* and the soybean phytopathogen *Fusarium virguliforme*^38^. In contrast, no inhibition of *E. coli*, *B. subtilis* and *S. cerevisiae* in presence of _D_-Phe-_L_-Val-_D_-Val-_L_-Tyr or _D_-Phe-_L_-Val-_D_-Val-_L_-Phe was observed^39^.

The antifungal activity of the tryptoquialanines was also evaluated, since these compounds seemed to be a chemical response of *P. digitatum* against *P. citrinum* in the chemical warfare. Tryptoquialanines **1** and **4** were tested and revealed an antifungal activity against *P. citrinum*. Compounds **1** and **4** had MIC of 300 µg mL^−1^, inhibiting *P. citrinum* spore production (Fig. S36). To the best of our knowledge, it is the first report of an antimicrobial activity of the tryptoquialanines. Recently, **1** was demonstrated as an insecticidal compound against *Ae. aegypti* larvae^23^; the antifungal activity can provide more understanding about the role of the tryptoquialanines in the citrus-pathogen environment once these compounds are not required for *P. digitatum* virulence^34^.

### Chemical warfare alters *P. digitatum* cell wall in co-culture

**Figure 6:**
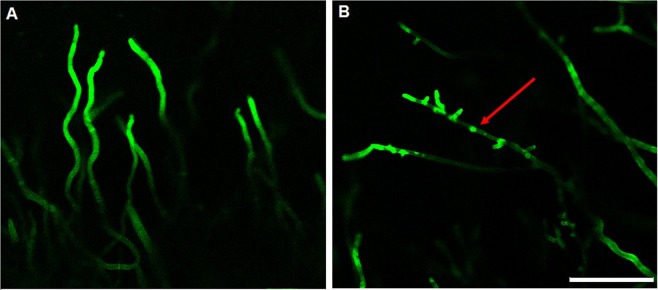
Confocal laser scanning microscopy of Congo Red-stained *P. digitatum* hyphae (**A**) distant from *P. citrinum* and (**B**) in the zone of confrontation. Patches of Congo Red indicates a defective fungal cell wall. Bars = 5.0 µm.

To investigate the action of the secondary metabolites during the fungal interaction, co-culture samples were stained with Congo Red and observed through confocal laser scanning microscopy. Congo Red is commonly used to stain polysaccharides containing β 1,4 linkages as, by example, the fungal cell wall component chitin^45,46^. *P. digitatum* hyphae were observed in the confrontation zone (sample) and compared with hyphae distant to the interface region (control) (Fig. S37). Control hyphae were homogeneous stained with Congo Red while hyphae in the interface region exhibited an altered staining pattern (Fig. 6), with irregular patches. Similar staining patterns were obtained for knockout mutant fungi which the deleted genes had a role in cell wall organization; as result, mutants exhibited defective cell walls and irregular staining^25,46,47^. Yet, abnormal staining with Calcofluor White was observed for *P. ostreatus* P89 treated with 36 °C; high temperature altered chitin distribution and cell wall integrity^48^.

**Figure 7:**
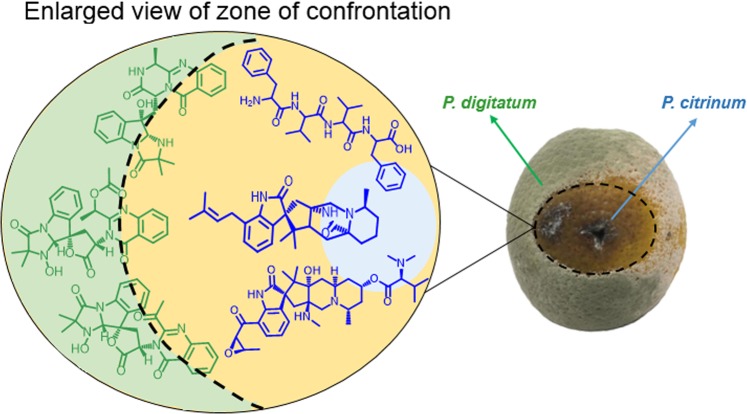
Chemical warfare between *P. citrinum* and *P. digitatum* in citrus fruit. Tryptoquialanines, citrinadins, chrysogenamide A, tetrapeptides and other metabolites are involved in the long-distance inhibition observed.

This data shows that *P. digitatum* hyphae, in contact with the metabolites diffused during the  co-culture, have a defective cell wall since Congo Red bounds to fungal cell wall structures. The fungal  cell wall is an attractive target of antimicrobials because they are not present in mammalian cells^49,50^. In conclusion, the microscopy analysis and the antifungal assays reinforce that the metabolites involved in the fungal interaction have potential as antifungal agents and may be the mechanism in nature that these phytopathogens developed to compete against other microorganisms for the host (Fig. 7).

## Conclusions

The search for new natural antimicrobials is a promising field in natural products research concerning the economic impact of postharvest diseases to worldwide agriculture. Furthermore, the appearance of fungi strains resistant to fungicides makes the discovery of new antifungal agents to replace synthetic compounds extremely important. Using co-cultivation between phytopathogens that compete for the same host, *P. digitatum* and *P. citrinum*, we observed a fungal interaction. Through MSI technique, we detected secondary metabolites diffused to the interface zone between the microorganisms. Tryptoquialanines, citrinadins, chyrsogenamide A and tetrapeptides exhibited great antifungal activity, confirming that  co-cultures and MSI technique are a good combination in the search of new natural antimicrobials.

Until this date, there has been no information about the interaction between citrus pathogenic fungi. Our data revealed compounds that play a role in the citrus microbial ecology. In addition, we demonstrated that the metabolites studied have great potential as antifungal agents since fungal cell walls are one of the main targets of antifungal compounds. The use of the identified compounds as natural antifungals instead of synthetic fungicides should be further investigated. This paper opens new research possibilities and contributes to the environmental and human health, helping in the search of safer strategies for agriculture through the use of compounds obtained from natural sources.

## Supplementary information

Supplementary Information

## Data Availability

All data generated or analyzed during this study are included in this published article and its Supplementary Information file.
