# Supplementary material for: Antifungal potential of secondary metabolites involved in the interaction between citrus pathogens
Source: Sci Rep. 2019 Dec 9;9:18647. doi: 10.1038/s41598-019-55204-9 (PMC6901458; doi:10.1038/s41598-019-55204-9)
Supplement: Supplementary file 1 — Supplementary Information [file 41598_2019_55204_MOESM1_ESM.pdf]

SCIENTIFIC REPORTS

Antifungal potential of secondary metabolites involved in the interaction between citrus pathogens

Jonas Henrique Costa[1], Cristiane Izumi Wassano[1], Célio Fernando Figueiredo Angolini[2], Kirstin Scherlach[3], Christian Hertweck[3,4], Taícia Pacheco Fill[1*]

[1] Institute of Chemistry, University of Campinas, CP 6154, 13083-970, Campinas – SP, Brazil

[2] Center for Natural and Human Sciences, Federal University of ABC, 09210-580, Santo André – SP, Brazil

[3] Department of Biomolecular Chemistry, Leibniz Institute for Natural Product Research and Infection Biology – Hans Knöll Institute, Jena, Germany

[4] Chair of Natural Product Chemistry, Friedrich Schiller University Jena, 07743 Jena, Germany.

*Corresponding author: phone +55-19-35213092, e-mail taicia@unicamp.br

Supplementary Information

S1. Mass Spectrometry Imaging (MSI)

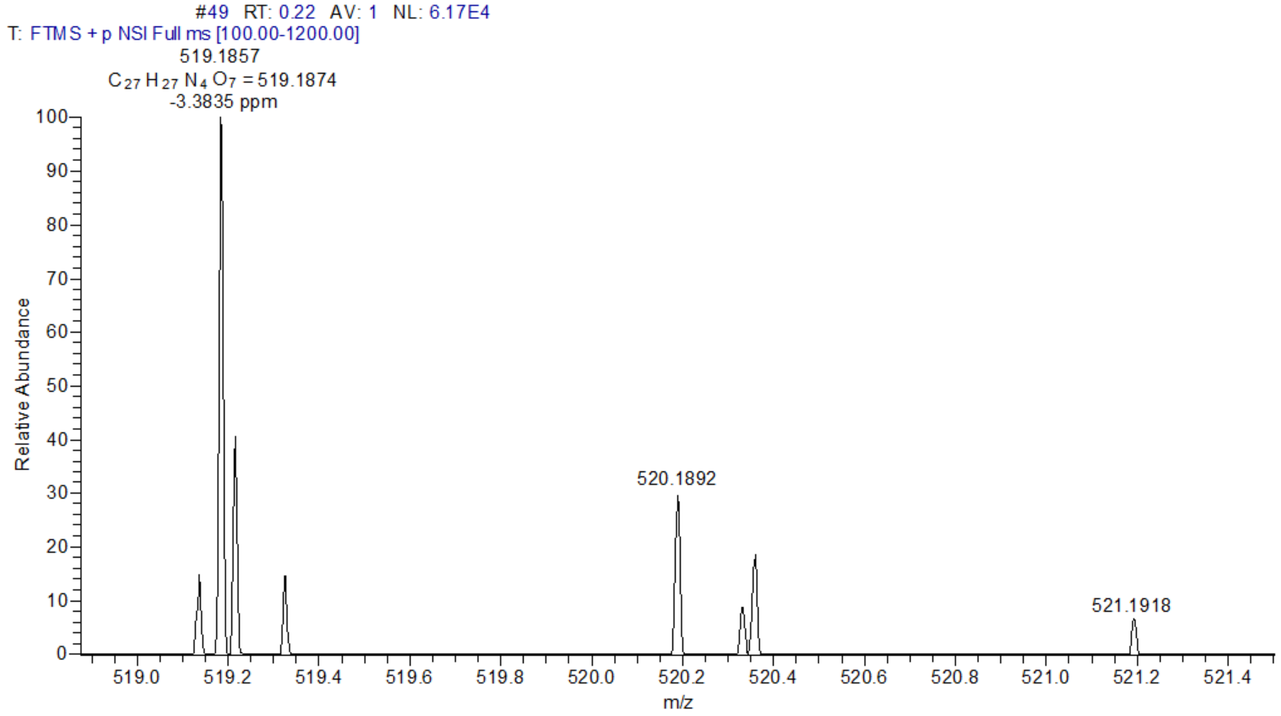

Figure S1. DESI-MS (+) signals obtained for tryptoquialanine A (**1**) on the surface of the *P. digitatum* and *P. citrinum* co-culture.

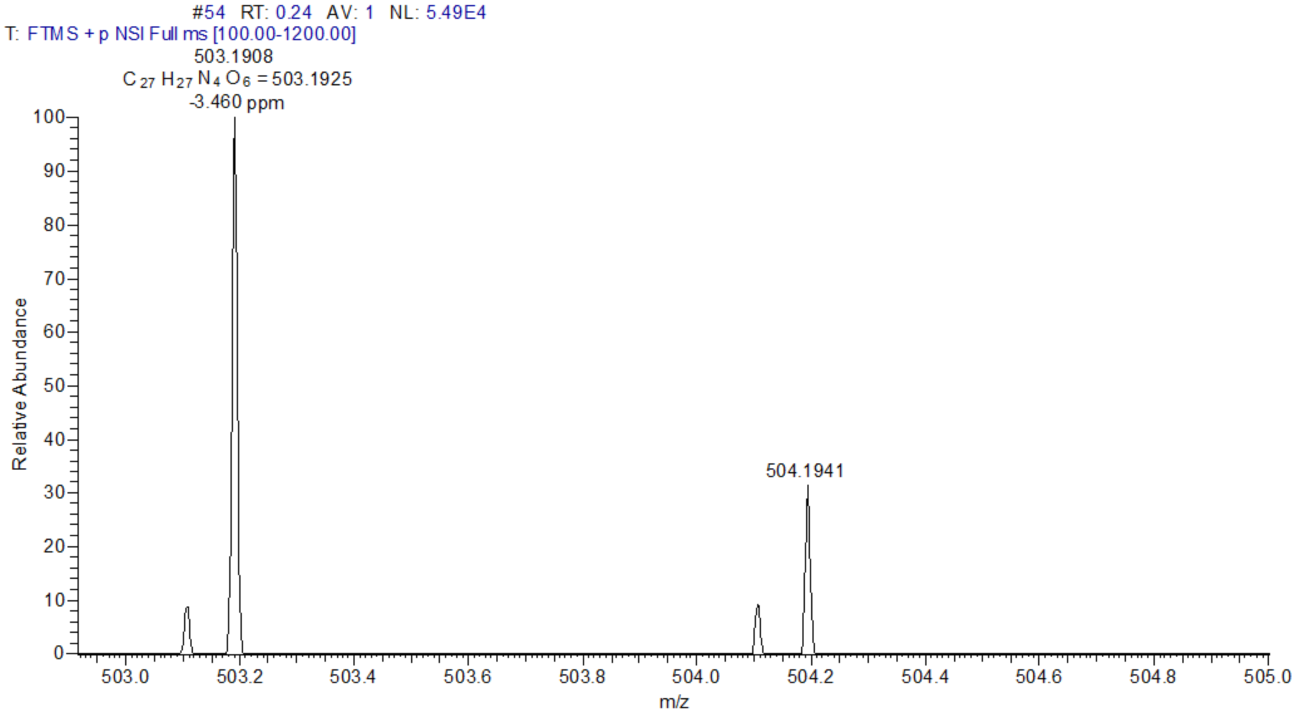

Figure S2. DESI-MS (+) signals obtained for tryptoquialanine C (**2**) on the surface of the *P. digitatum* and *P. citrinum* co-culture.

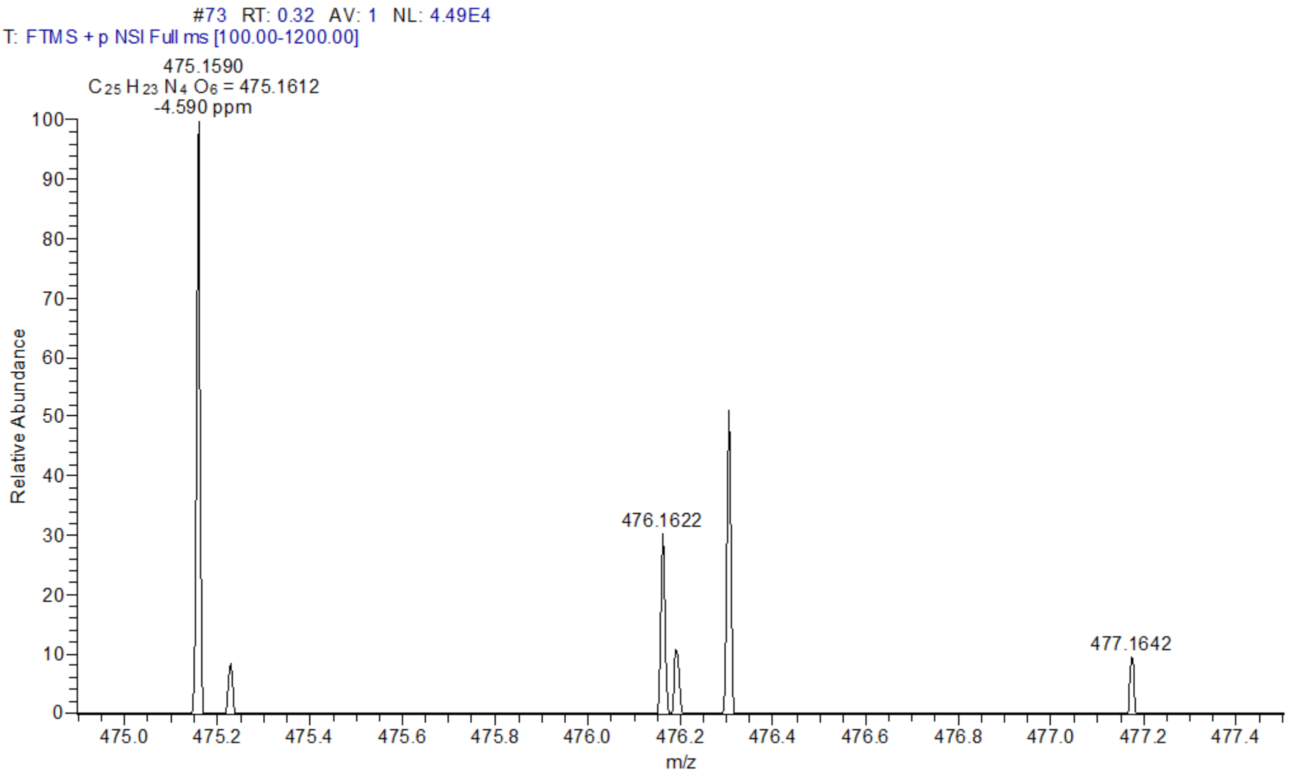

Figure S3. DESI-MS (+) signals obtained for tryptoquialanone (**3**) on the surface of the *P. digitatum* and *P. citrinum* co-culture.

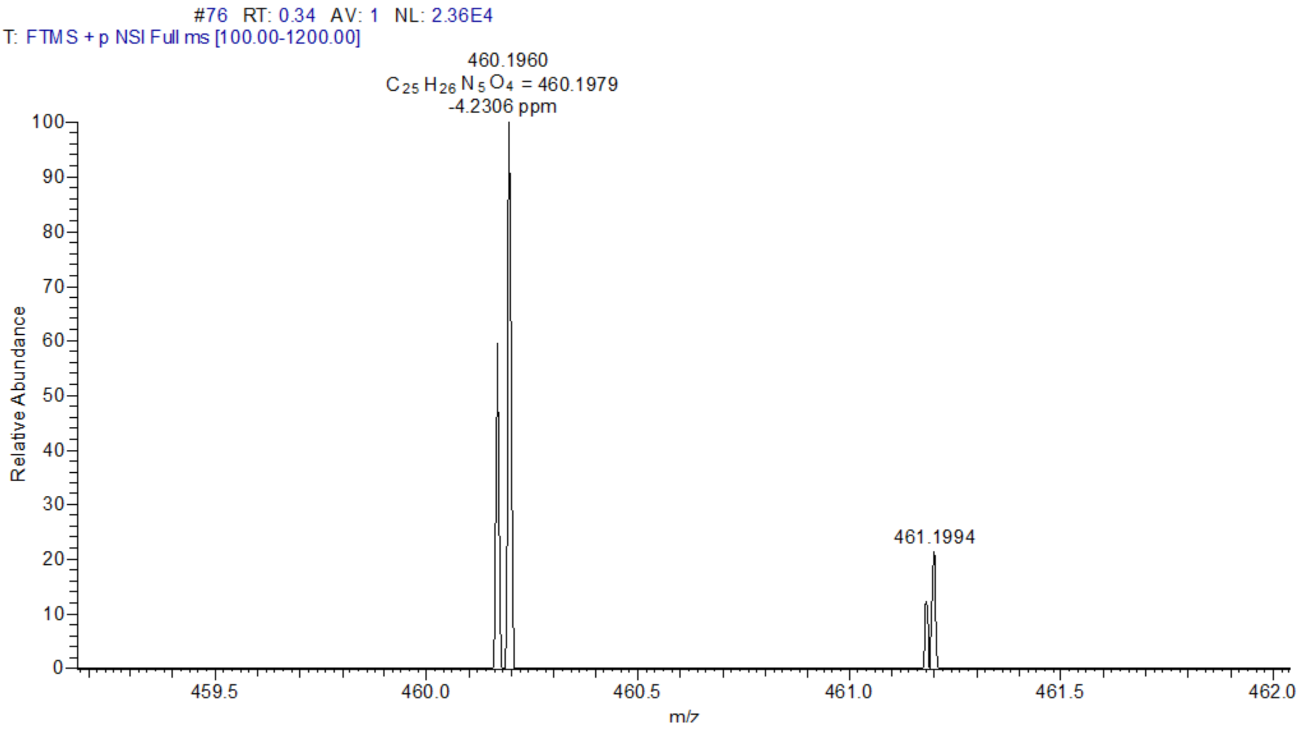

Figure S4. DESI-MS (+) signals obtained for 15-dimethyl-2-epi-fumiquinazoline A (**4**) on the surface of the *P. digitatum* and *P. citrinum* co-culture.

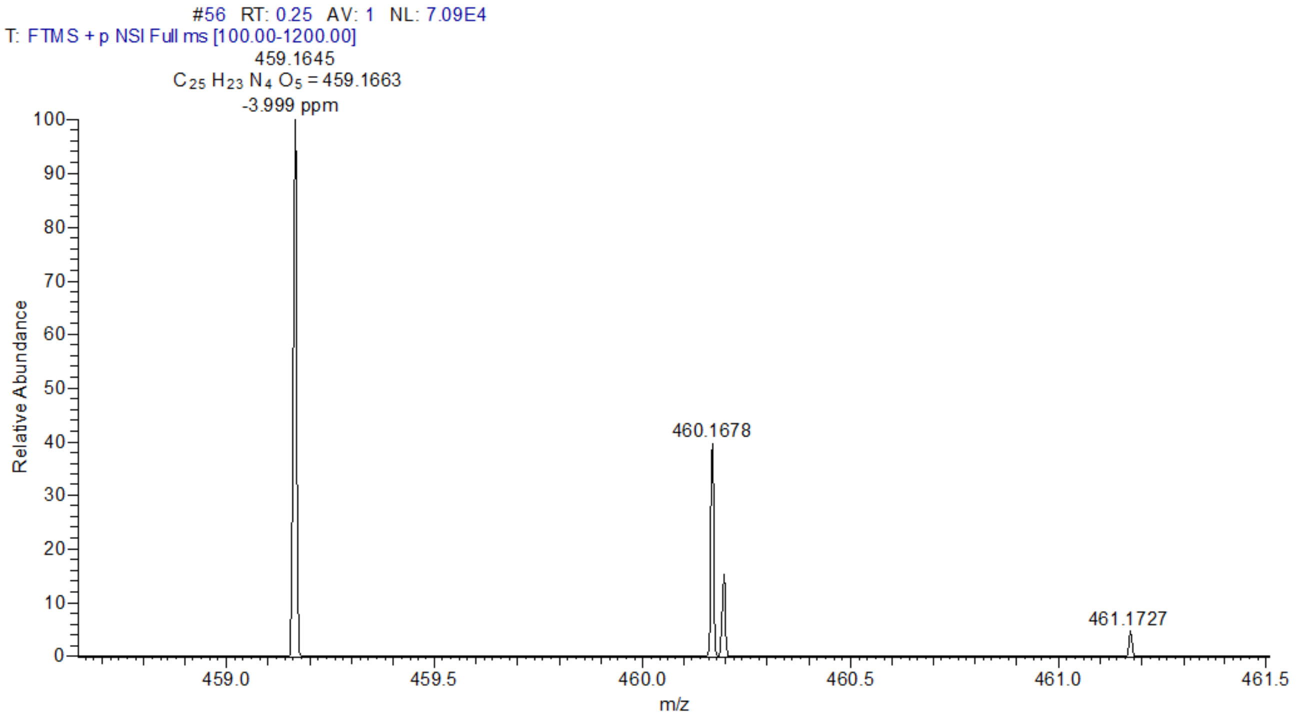

Figure S5. DESI-MS (+) signals obtained for deoxytryptoquialanone (**5**) on the surface of the *P. digitatum* and *P. citrinum* co-culture**.**

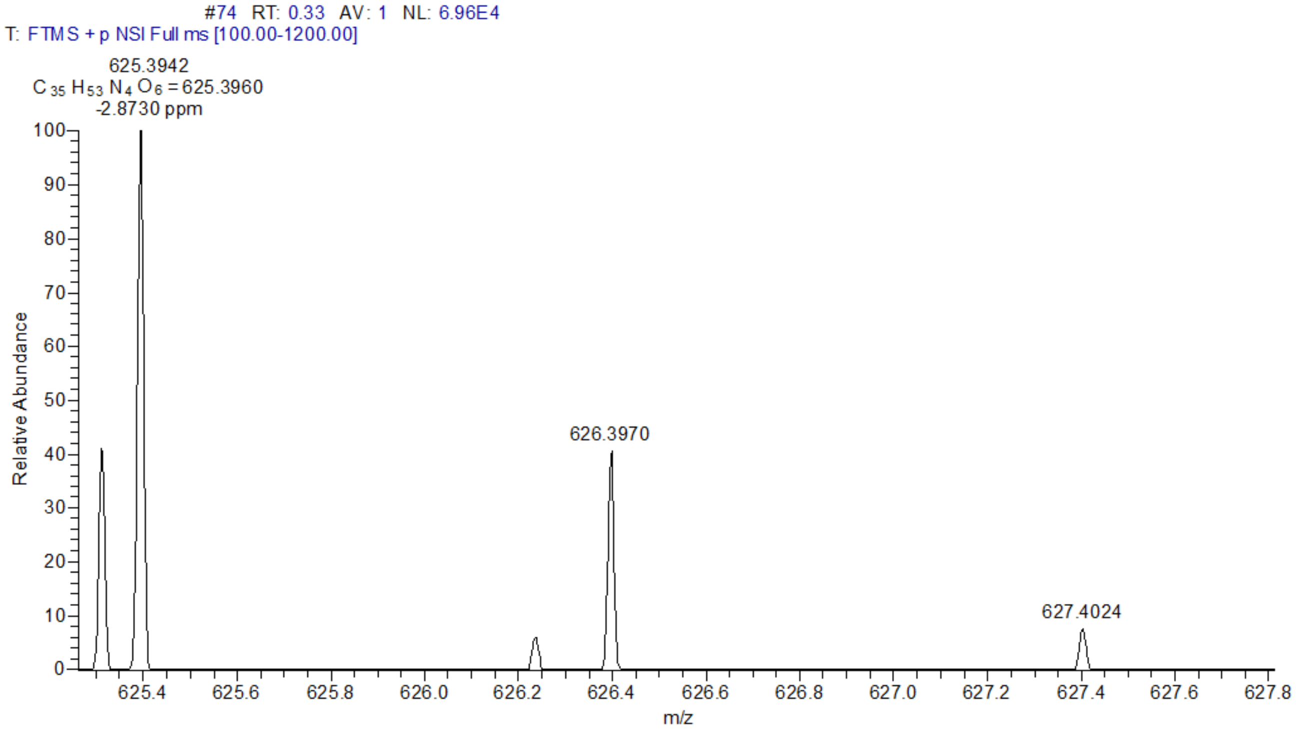

Figure S6. DESI-MS (+) signals obtained for citrinadin A (**6**) on the surface of the *P. digitatum* and *P. citrinum* co-culture**.**

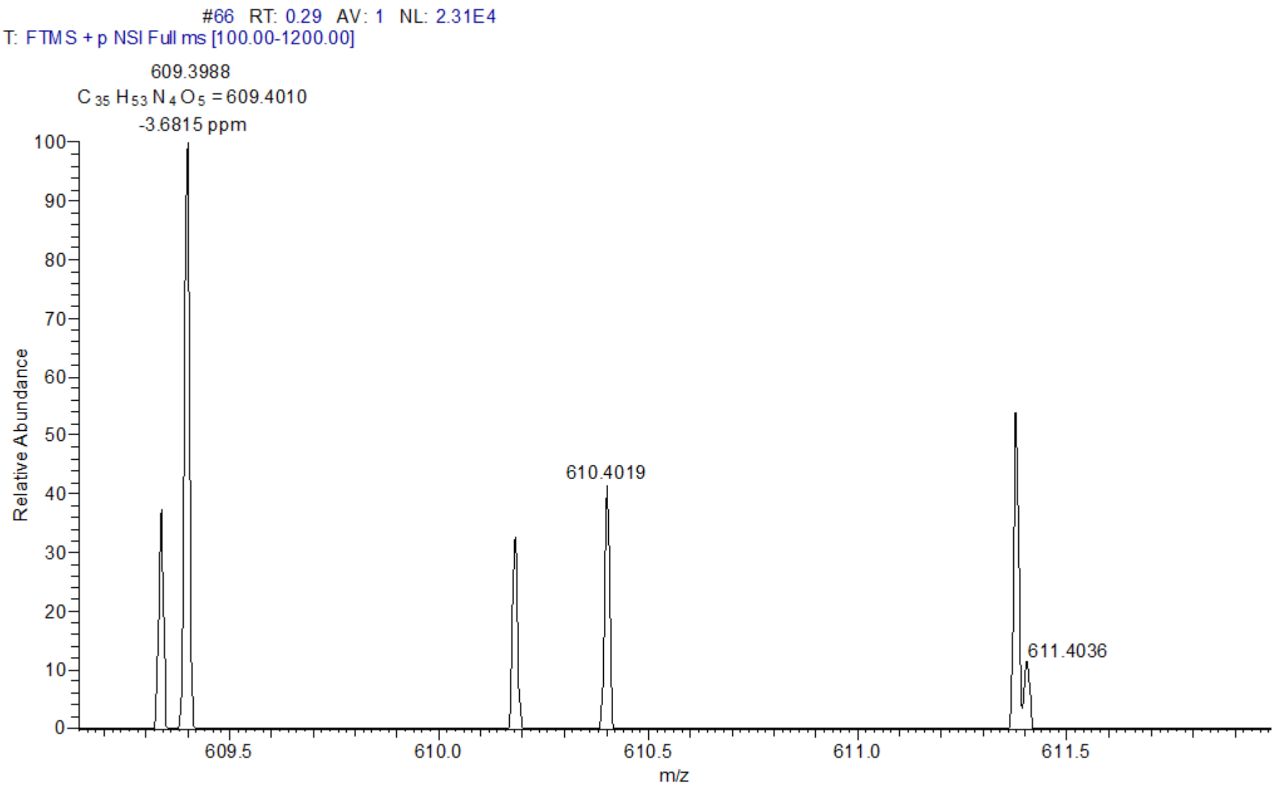

Figure S7. DESI-MS (+) signals obtained for deoxycitrinadin A (**7**) on the surface of the *P. digitatum* and *P. citrinum* co-culture**.**

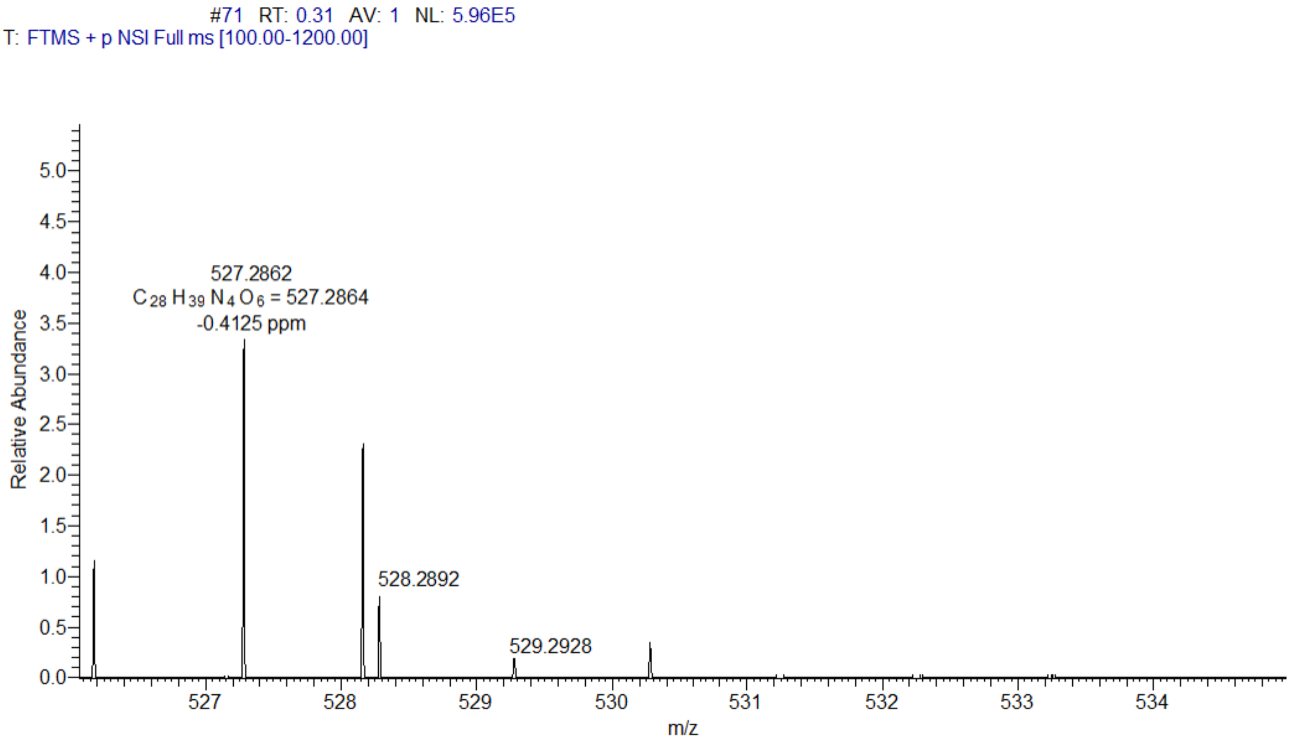

Figure S8. DESI-MS (+) signals obtained for Phe-Val-Val-Tyr (**8**) on the surface of the *P. digitatum* and *P. citrinum* co-culture**.**

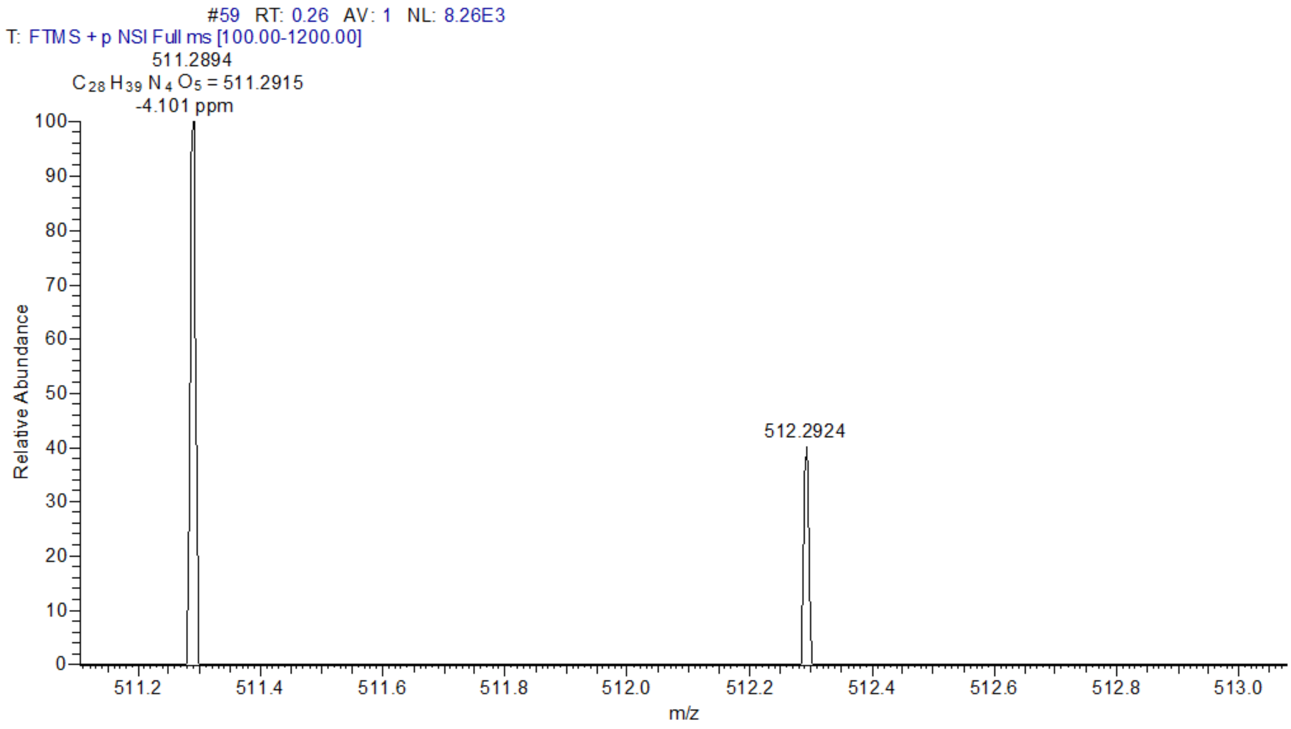

Figure S9. DESI-MS (+) signals obtained for Phe-Val-Val-Phe (**9**) on the surface of the *P. digitatum* and *P. citrinum* co-culture**.**

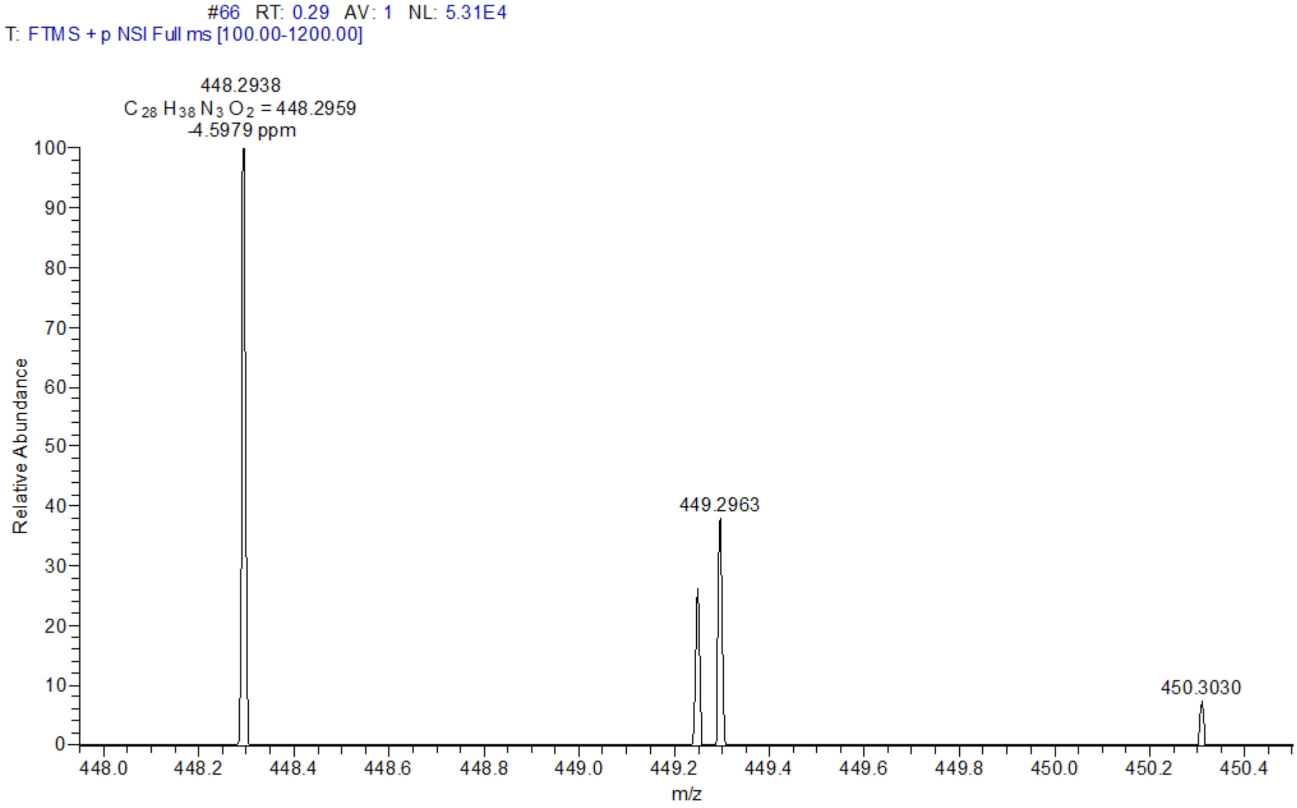

Figure S10. DESI-MS (+) signals obtained for chrysogenamide A (**10**) on the surface of the *P. digitatum* and *P. citrinum* co-culture**.**

S2. Mass Spectrometry of *in vivo* co-culture

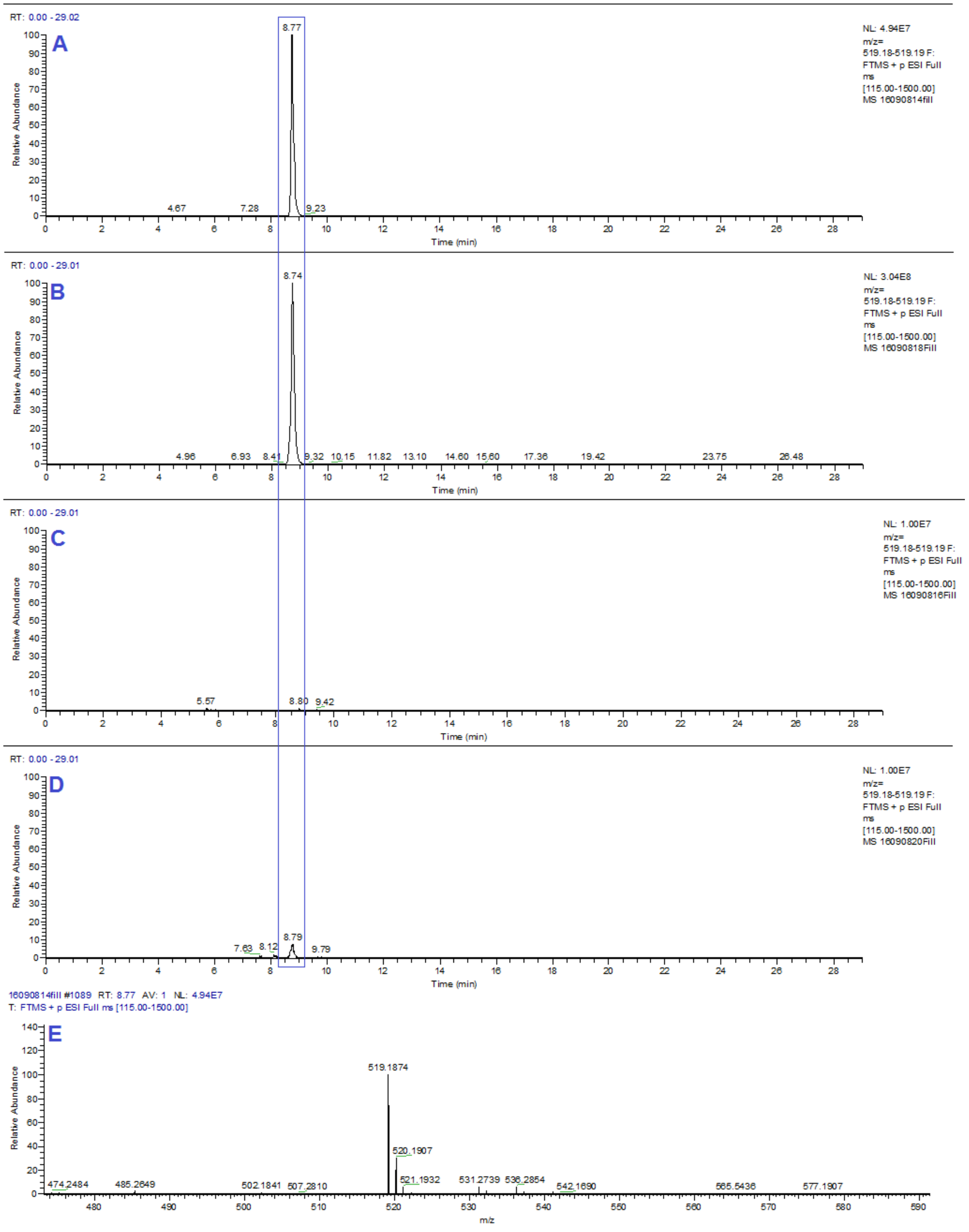

Figure S11. Extracted ion chromatograms of $[M+H]^+$ *m/z* 519.18, tryptoquialanine A (**1**), for *in vivo* extracts of (A) co-culture, (B) *P. digitatum*, (C) *P. citrinum* and in (D) orange control. (E) Mass spectrum of **1**.

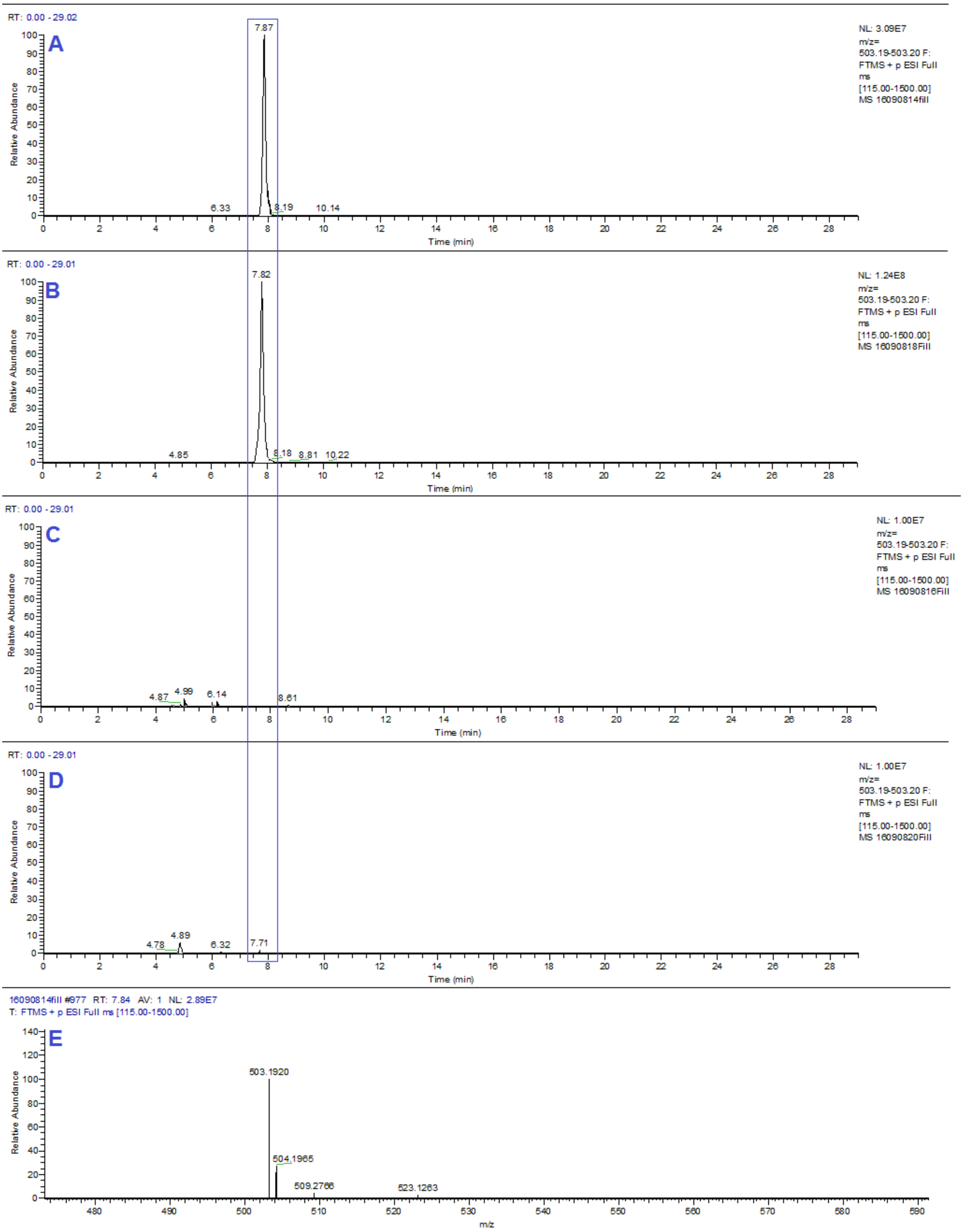

Figure S12. Extracted ion chromatograms of $[M+H]^+$ *m/z* 503.19, tryptoquialanine C (**2**), for *in vivo* extracts of (A) co-culture, (B) *P. digitatum*, (C) *P. citrinum* and in (D) orange control. (E) Mass spectrum of **2**.

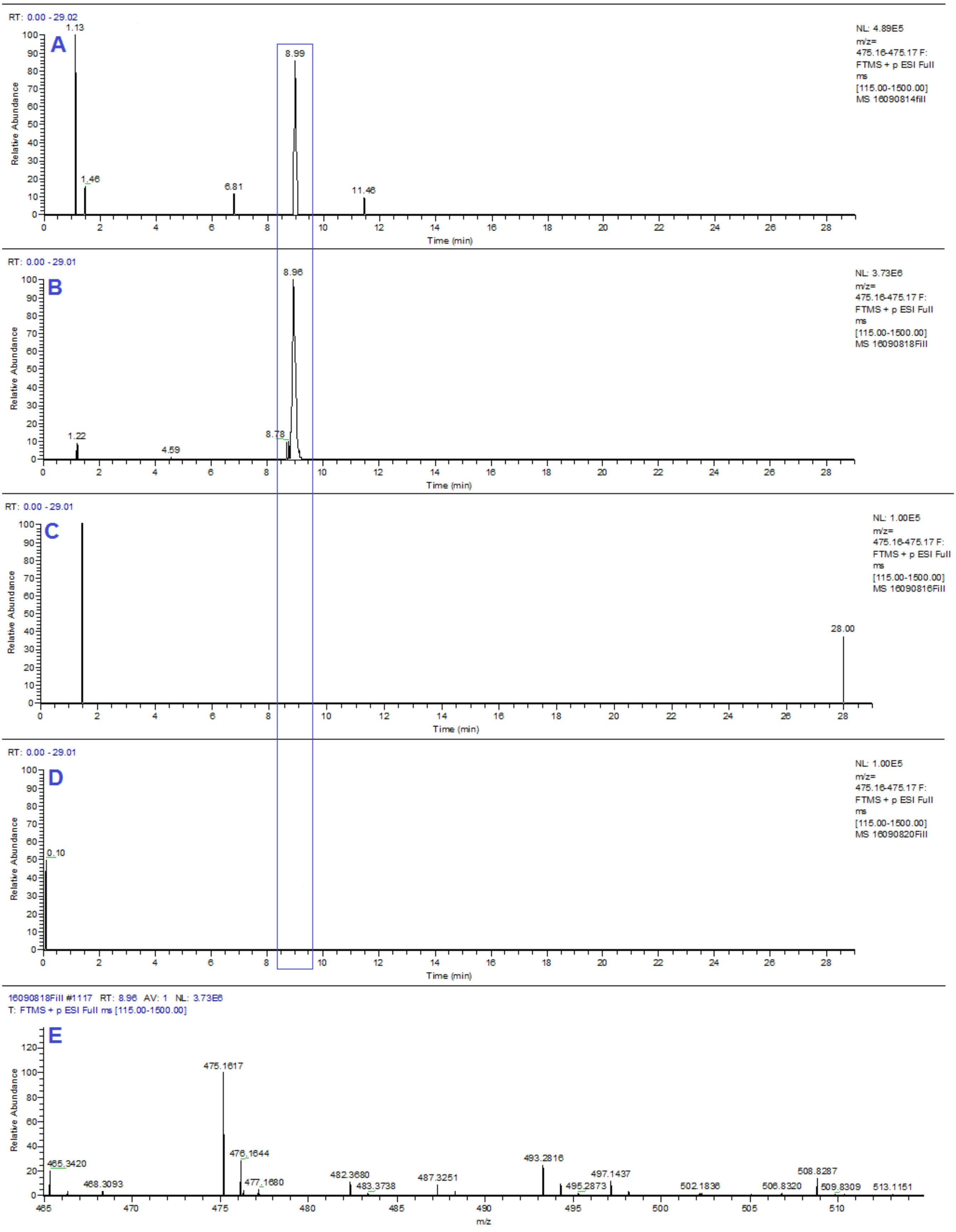

Figure S13. Extracted ion chromatograms of $[M+H]^+$ *m/z* 475.16, tryptoquialanone (**3**), for *in vivo* extracts of (A) co-culture, (B) *P. digitatum*, (C) *P. citrinum* and in (D) orange control. (E) Mass spectrum of **3**.

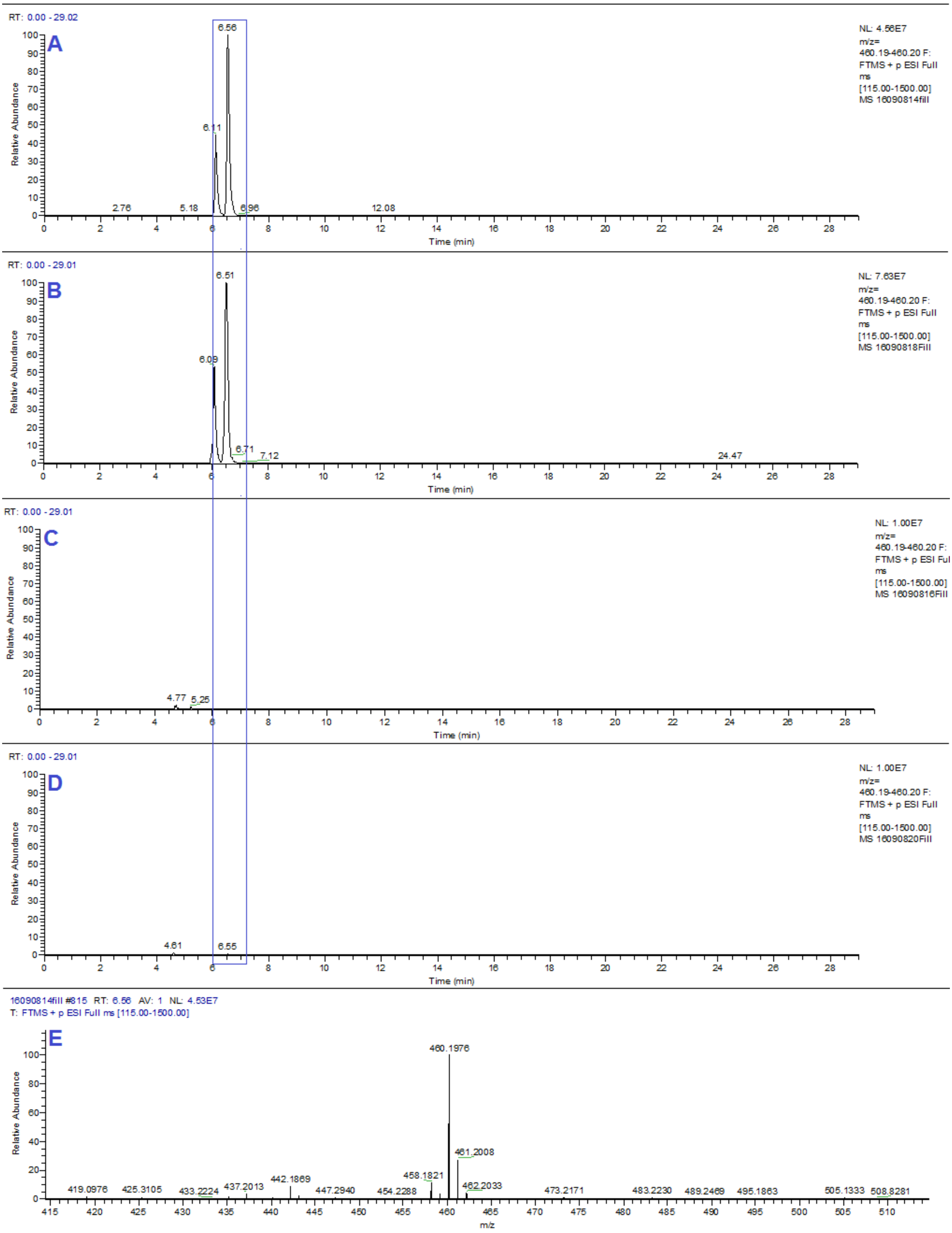

Figure S14. Extracted ion chromatograms of $[M+H]^+$ *m/z* 460.19, 15-dimethyl-2-epi-fumiquinazoline A (**4**), for *in vivo* extracts of (A) co-culture, (B) *P. digitatum*, (C) *P. citrinum* and in (D) orange control. (E) Mass spectrum of **4**.

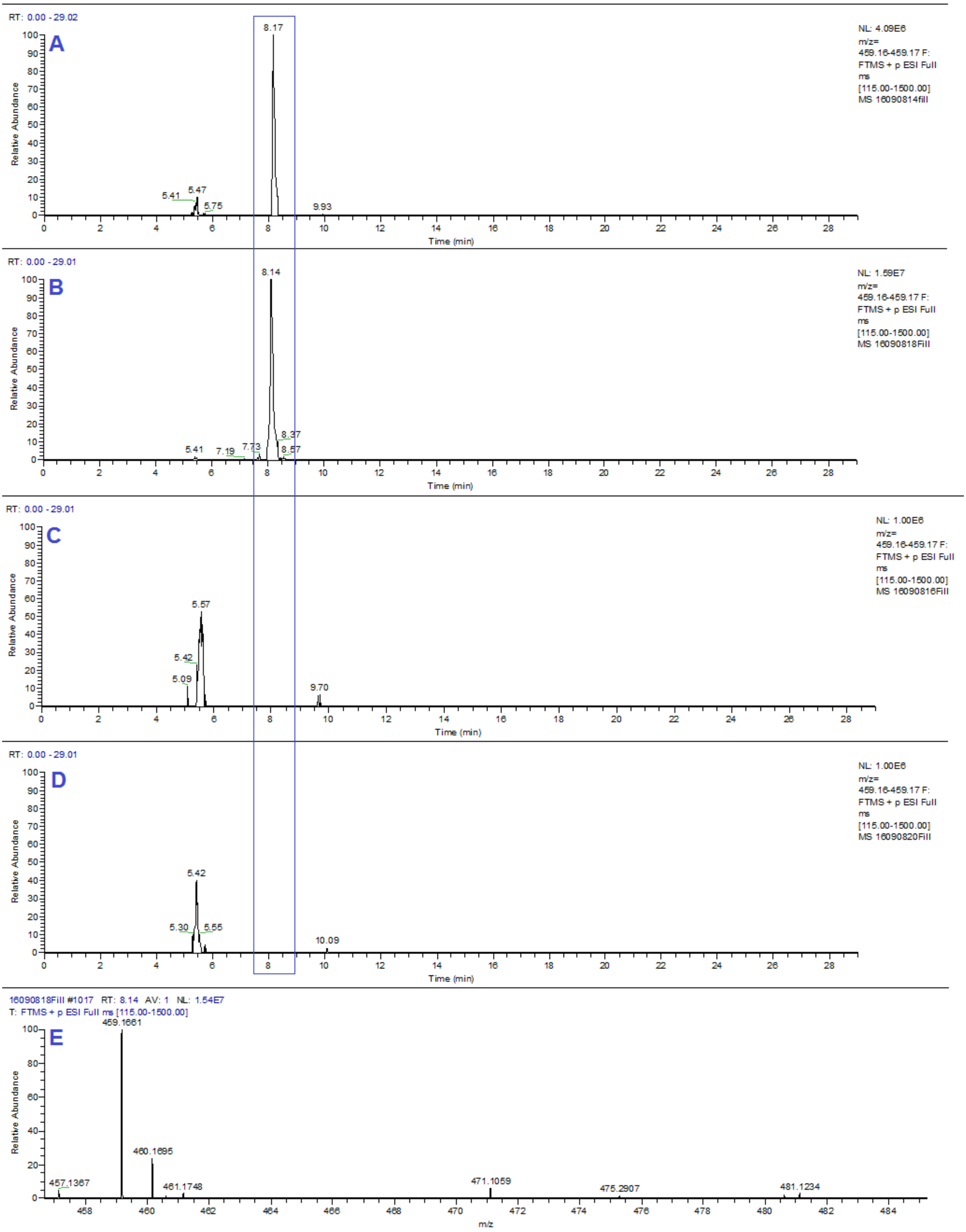

Figure S15. Extracted ion chromatograms of $[M+H]^+$ *m/z* 459.16, deoxytryptoquialanone (**5**), for *in vivo* extracts of (A) co-culture, (B) *P. digitatum*, (C) *P. citrinum* and in (D) orange control. (E) Mass spectrum of **5**.

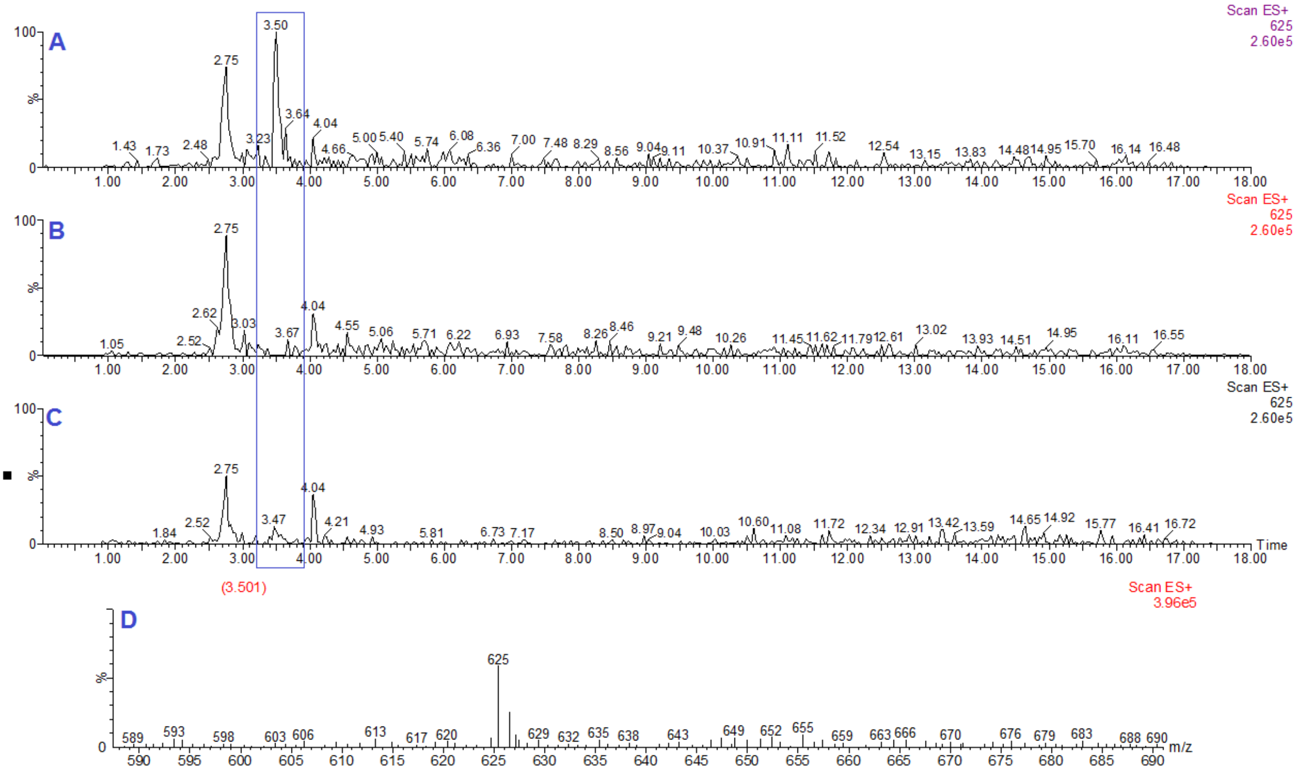

Figure S16. Extracted ion chromatograms of $[M+H]^+$ *m/z* 625, citrinadin A (**6**), for *in vivo* extracts of (A) co-culture and (B) *P. citrinum* and in (C) orange control. (D) Mass spectrum of **6**.

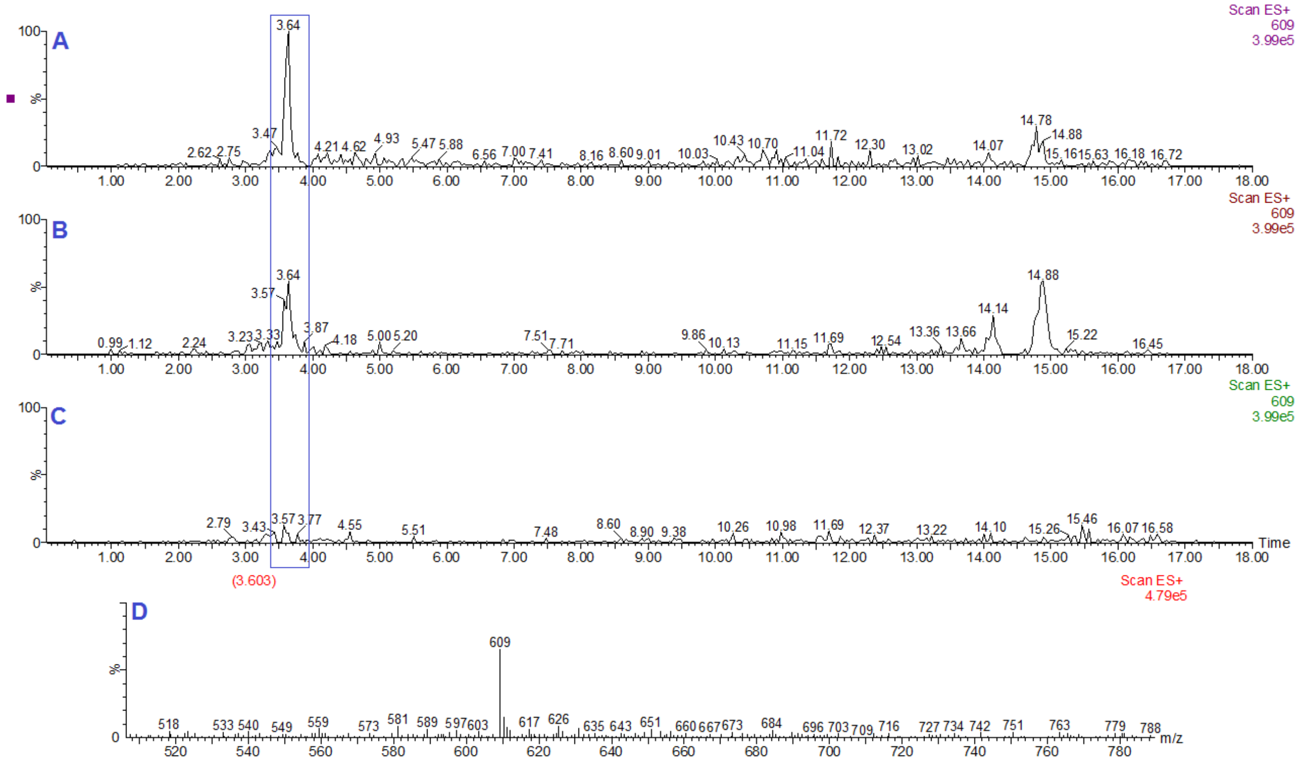

Figure S17. Extracted ion chromatograms of $[M+H]^+$ *m/z* 609, deoxycitrinadin A (**7**), for *in vivo* extracts of (A) co-culture and (B) *P. citrinum* and in (C) orange control. (D) Mass spectrum of **7**.

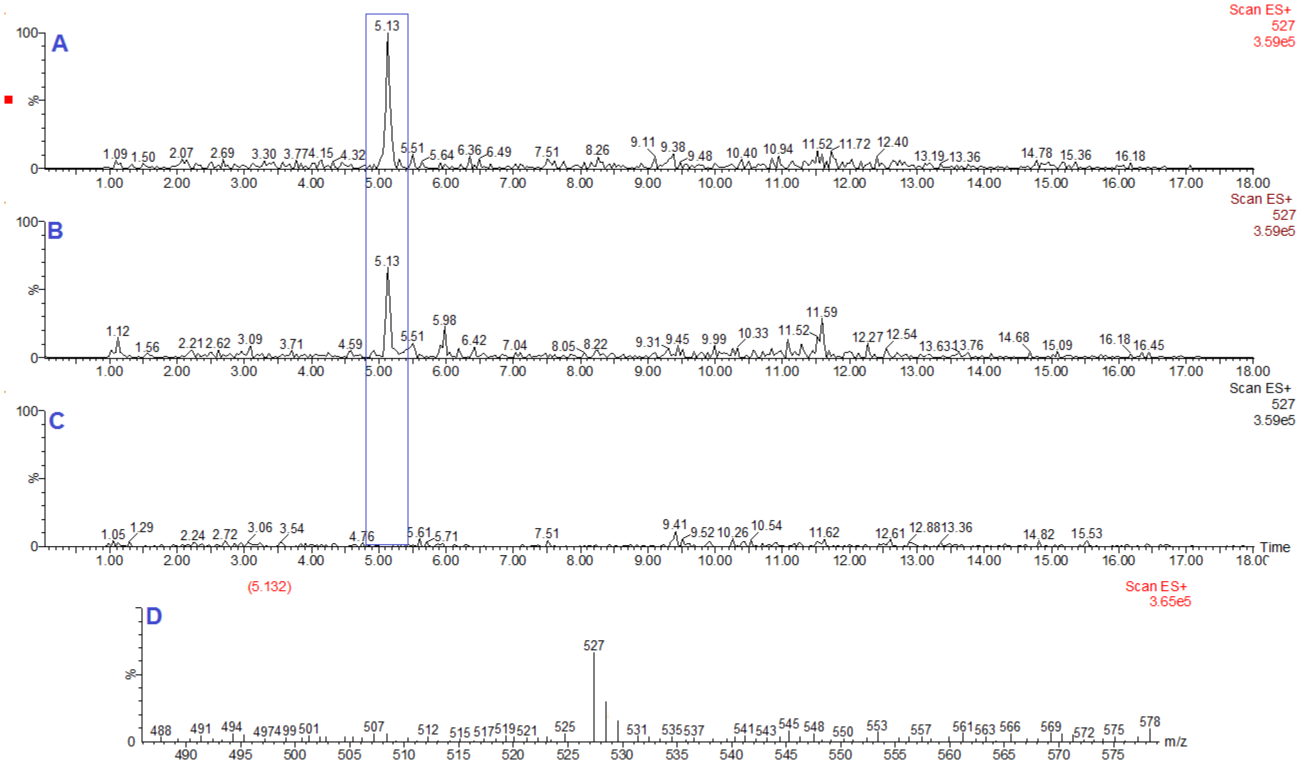

Figure S18. Extracted ion chromatograms of $[M+H]^+$ *m/z* 527, Phe-Val-Val-Tyr (**8**), for *in vivo* extracts of (A) co-culture and (B) *P. citrinum* and in (C) orange control. (D) Mass spectrum of **8**.

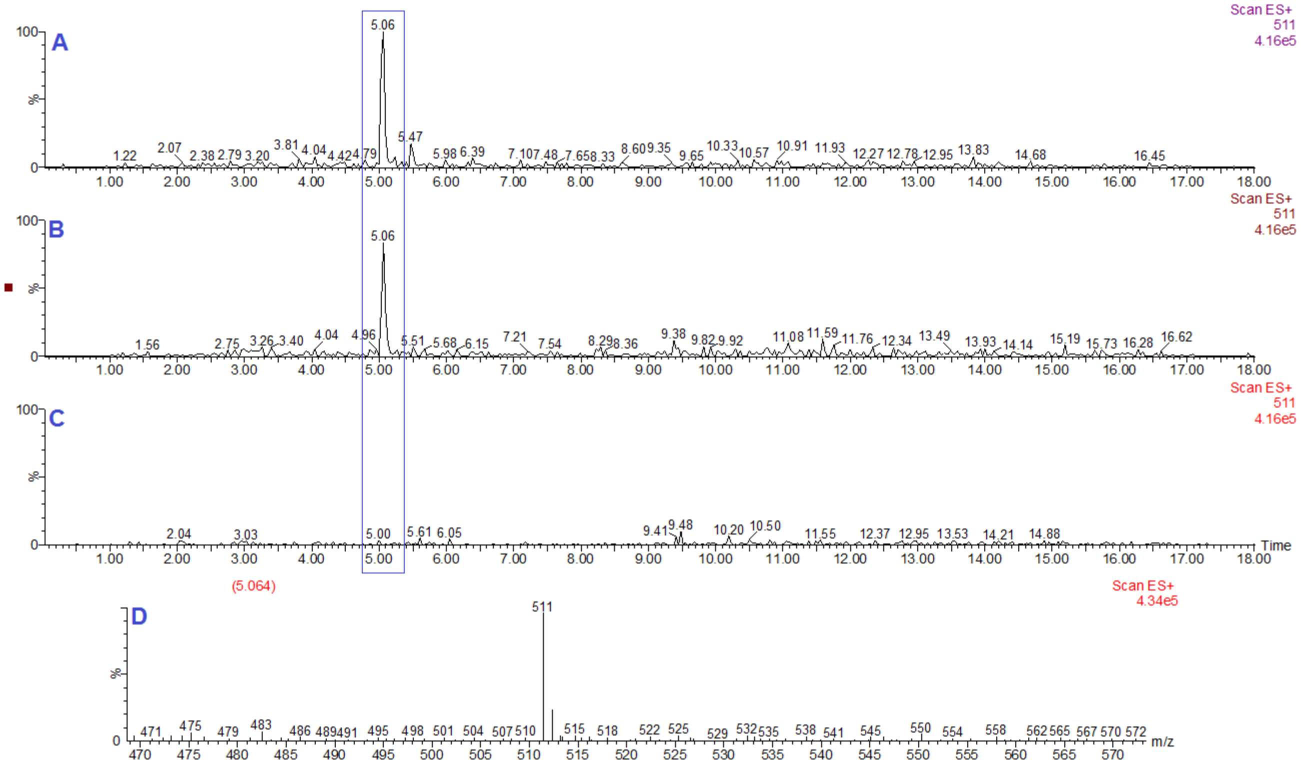

Figure S19. Extracted ion chromatograms of $[M+H]^+$ *m/z* 511, Phe-Val-Val-Phe (**9**), for *in vivo* extracts of (A) co-culture and (B) *P. citrinum* and in (C) orange control. (D) Mass spectrum of **9**.

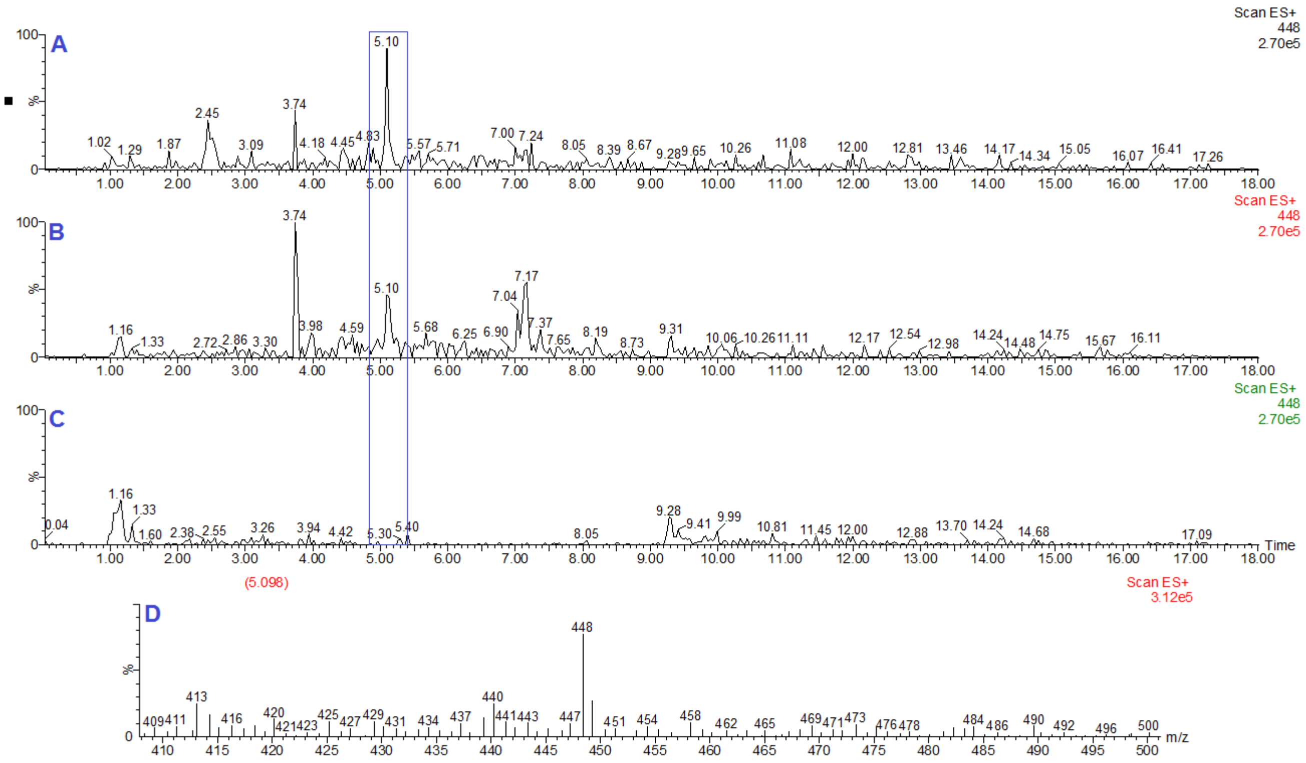

Figure S20. Extracted ion chromatograms of [M+H]$^+$ *m/z* 448, chrysogenamide A (**10**), for *in vivo* extracts of (A) co-culture and (B) *P. citrinum* and in (C) orange control. (D) Mass spectrum of **10**.

S3. Characterization of secondary metabolites

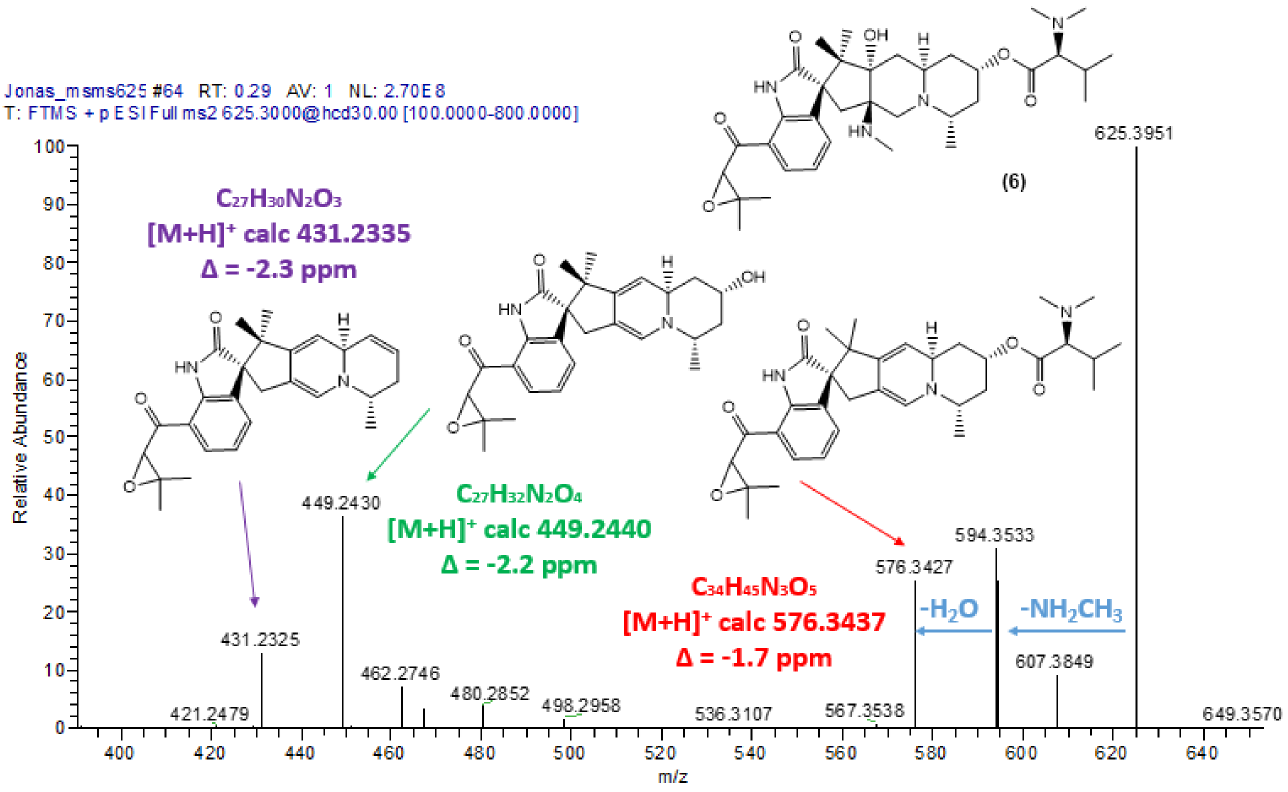

Figure S21. MS/MS spectrum of citrinadin A (**6**) (30 eV).

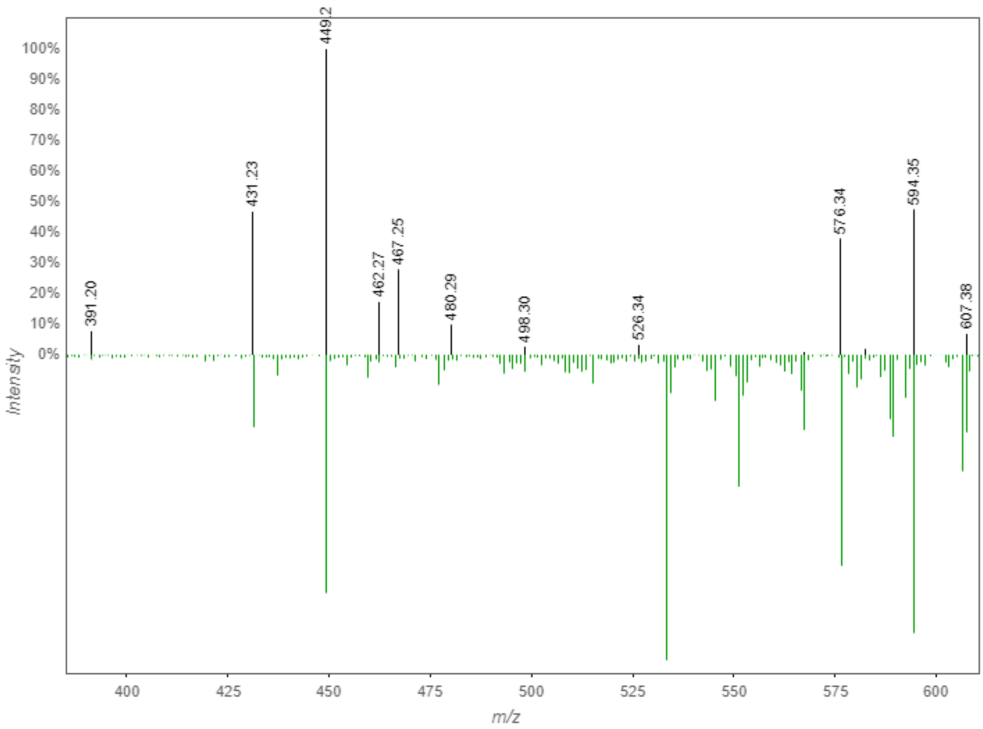

Figure S22. MS/MS match between GNPS database (green) and ion at *m/z* 625 isolated from co-culture extract (black). The tandem mass spectrum shared 5 mass fragments in common (*m/z* 607.38, 594.35, 576.34, 449.24 and 431.23), suggesting that ion at *m/z* 625 could be citrinadin A.

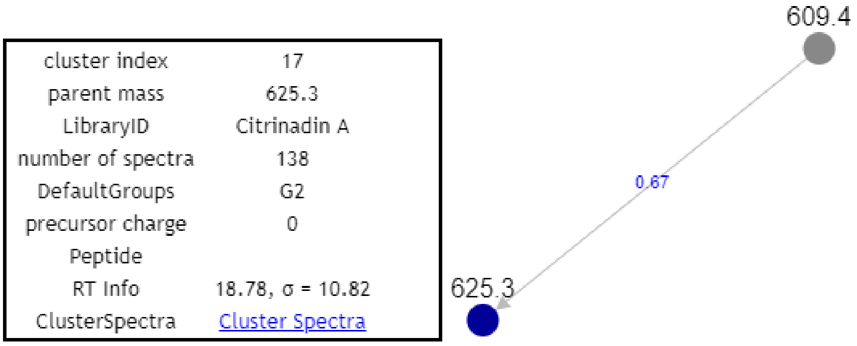

Figure S23. MS/MS molecular networking analysis of compounds isolated from *P. citrinum* and *P. digitatum* co-culture extract. The blue node represents a match between *m/z* 625 and citrinadin A in GNPS database. The grey (m/z 609) and blue node are related metabolites grouped in same cluster with a cosine of 0.67.

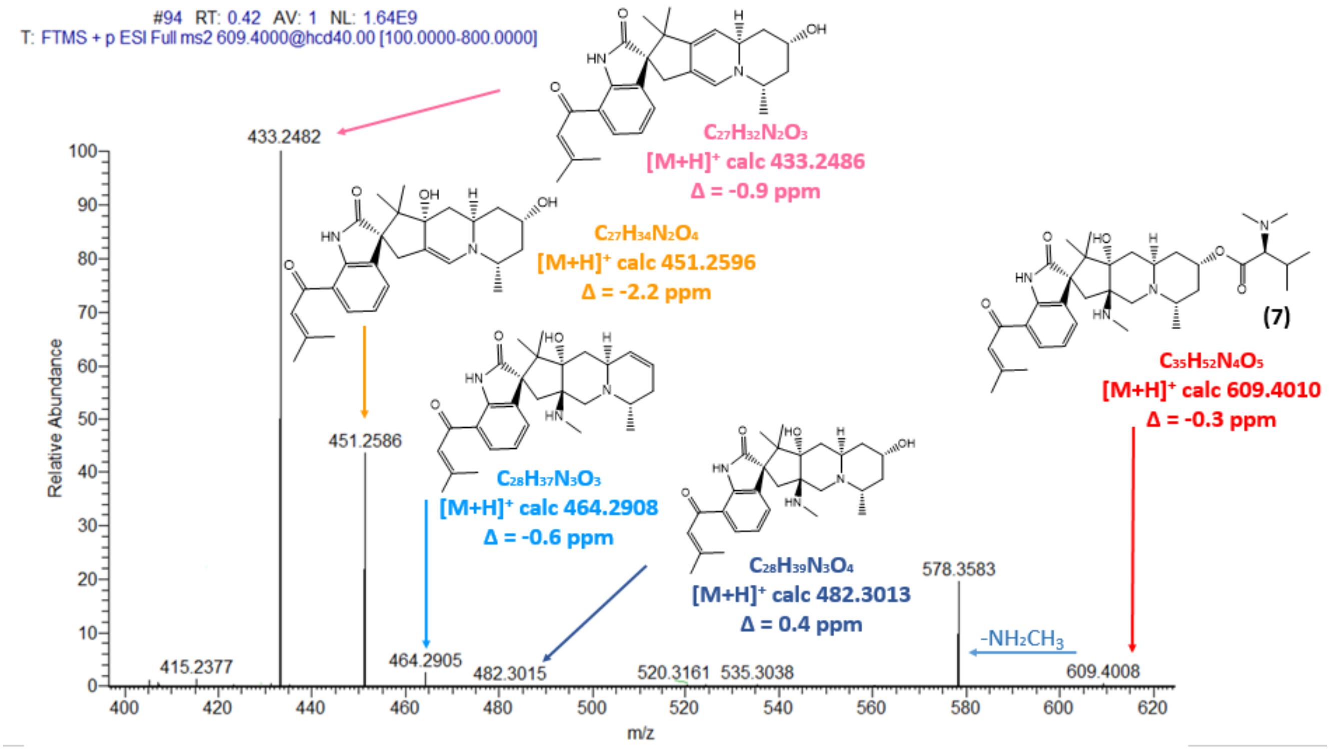

Figure S24. MS/MS spectrum of deoxycitrinadin A (**7**) (40 eV) and proposed fragmentation structures.

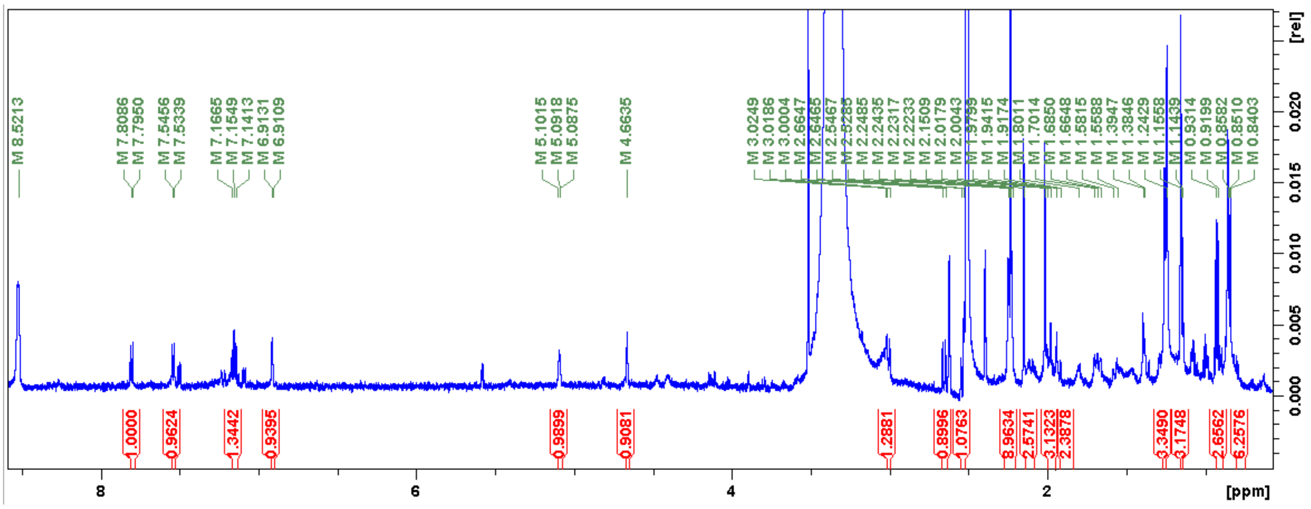

Figure S25. ^{1}H NMR spectrum of Deoxycitrinadin A (**7**) (600.17 MHz, DMSO).

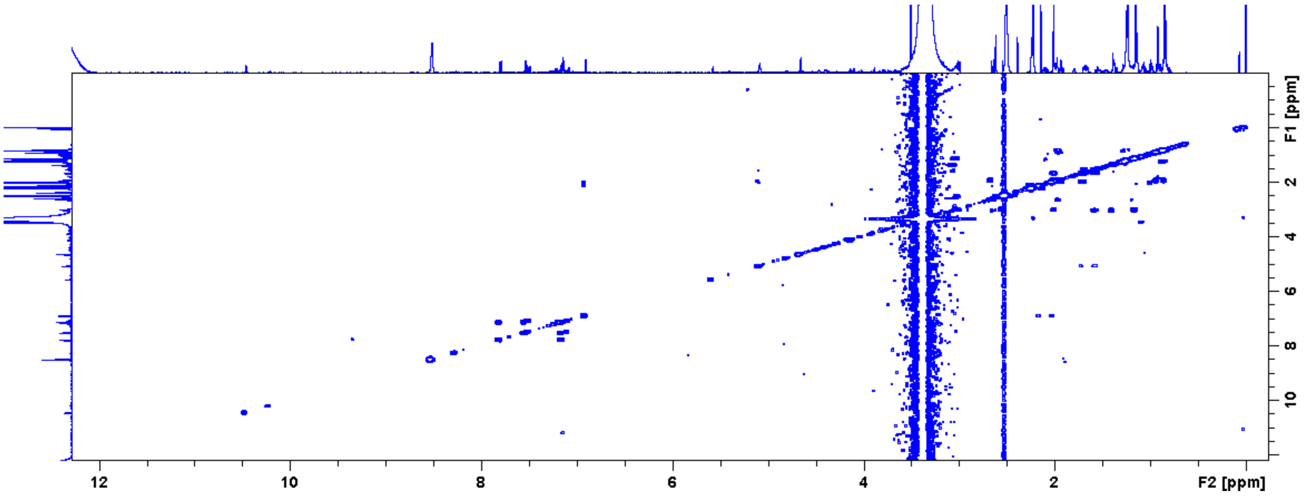

Figure S25. COSY NMR spectrum of Deoxycitrinadin A (**7**) (600.17 MHz, DMSO).

Table S1. ^{1}H NMR data and ^{1}H-^{1}H correlations in COSY for **7** (600 MHz, DMSO) and comparison with the literature (δ in ppm, *J* in Hz).

7

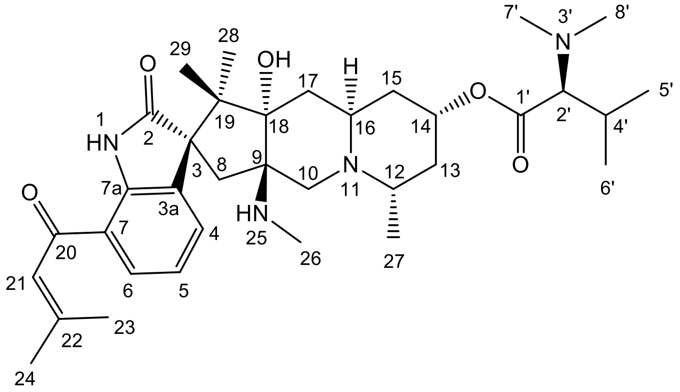

Position	^{1}H δ (m, *J*)	^{1}H δ (m, *J*)[1]	COSY
1	8.52 (s)	9.77 (s)	
4	7.54 (d, 7.0)	7.57 (d, 7.2)	H-5
5	7.15 (t, 7.0)	7.06 (dd, 7.8, 7.2)	H-4, H-6
6	7.79 (d, 8.1)	7.67 (dd, 7.8, 0.6)	H-5
8	1.98, 1.94 (ABq, 11.5)	2.08, 2.04 (ABq, 14.4)	
10	3.00 (d, 10.9)	3.15 (d, 10.8)	
10	2.53 (d, 10.9)	2.54 (d, 10.8)	
12	3.03 – 2.99 (m)	3.01 (quin, 6.6)	H-27, H-13
13	1.58 – 1.55 (m)	1.50 – 1.45 (m)	H-12, H-14
13	2.03 – 1.99 (m)	2.04 – 1.98 (comp)	
14	5.10 – 5.08 (m)	5.19 – 5.20 (m)	H-13, H-15
15	1.70 – 1.66 (m)	1.80 – 1.76 (comp)	H-14, H-16
15	1.81 – 1.79 (m)	1.80 – 1.76 (comp)	
16	3.18 – 3.17 (m)	3.20 – 3.14 (m)	H-15, H-17
17	1.39 – 1.38 (m)	1.36 – 1.31 (comp)	H-16
17	1.58 – 1.55 (m)	1.53 (dd, 13.2, 4.2)	
18-OH	4.66 (s)	4.70 (d, 2.4)	
21	6.91 – 6.90 (m)	6.75 – 6.74 (m)	H-24, H-23
23	2.15 (s)	2.17 (d, 1.2)	H-21
24	2.01 (s)	2.01 (d, 1.2)	H-21
26	2.24 – 2.23 (m)	2.28 (s)	
27	1.15 (d, 7.1)	1.19 (d, 7.2)	H-12
28	0.85 (s)	0.96 (s)	
29	1.24 (s)	1.36 – 1.31 (comp)	
2’	2.65 (d, 10.9)	2.68 (d,10.8)	H-4’
4’	1.95 – 1.93 (m)	2.04 – 1.98 (comp)	H-2’, H-5’, H-6’
5’	0.92 (d, 6.9)	0.95 (d, 6.6)	4’
6’	0.85 (d, 6.4)	0.88 (d, 6.6)	4’
7’	2.23 (s)	2.29 (s)	
8’	2.23 (s)	2.29 (s)	

[1] Bian, Z., Marvin, C. C., Martin, S. F. Enantioselective Total Synthesis of (-)-Citrinadin A and Revision of Its Stereochemical Structure. *J Am Chem Soc* **135**, 10886-10889, https://doi.org/10.1021/ja405547f (2013).

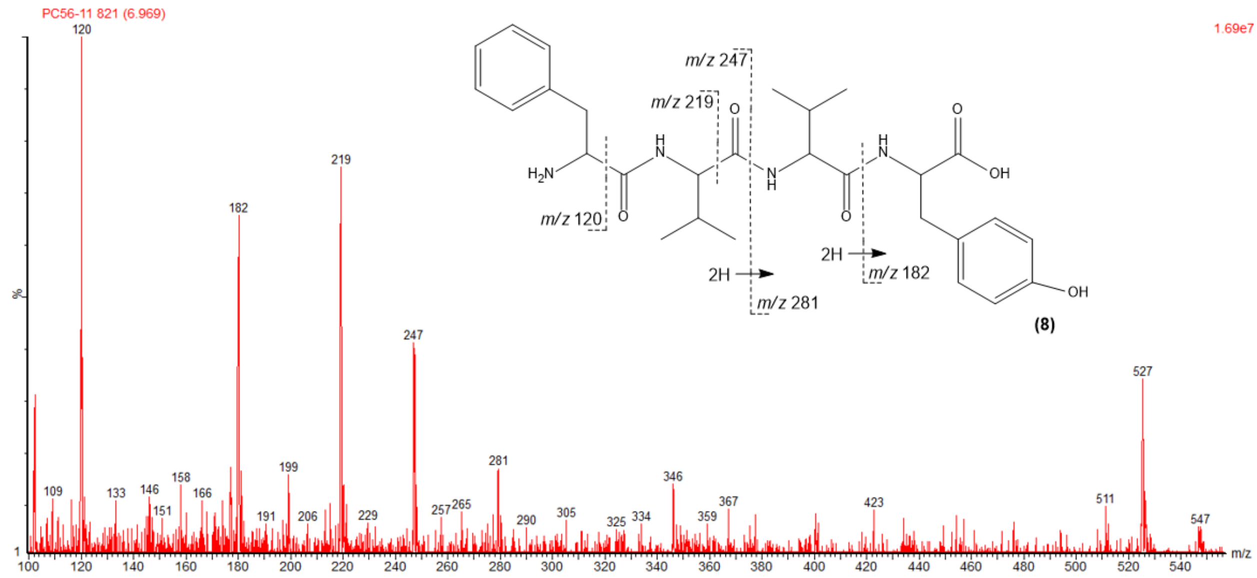

Figure S27. MS/MS spectrum of Tyr-Val-Val-Phe (**8**).

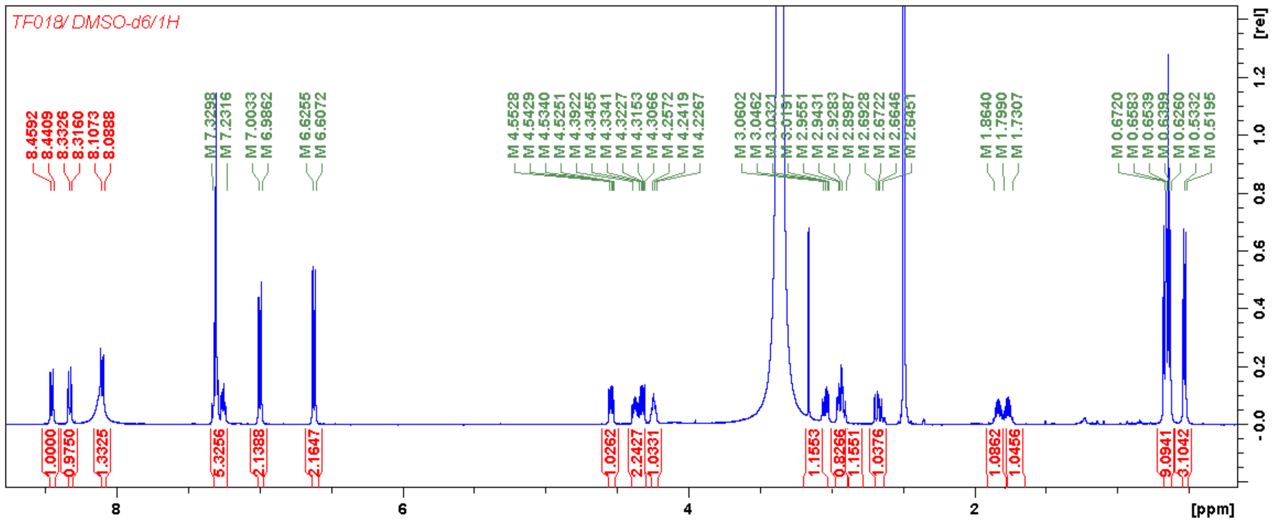

Figure S28. ^{1}H NMR spectrum of Tyr-Val-Val-Phe (**8**) (500.13 MHz, DMSO).

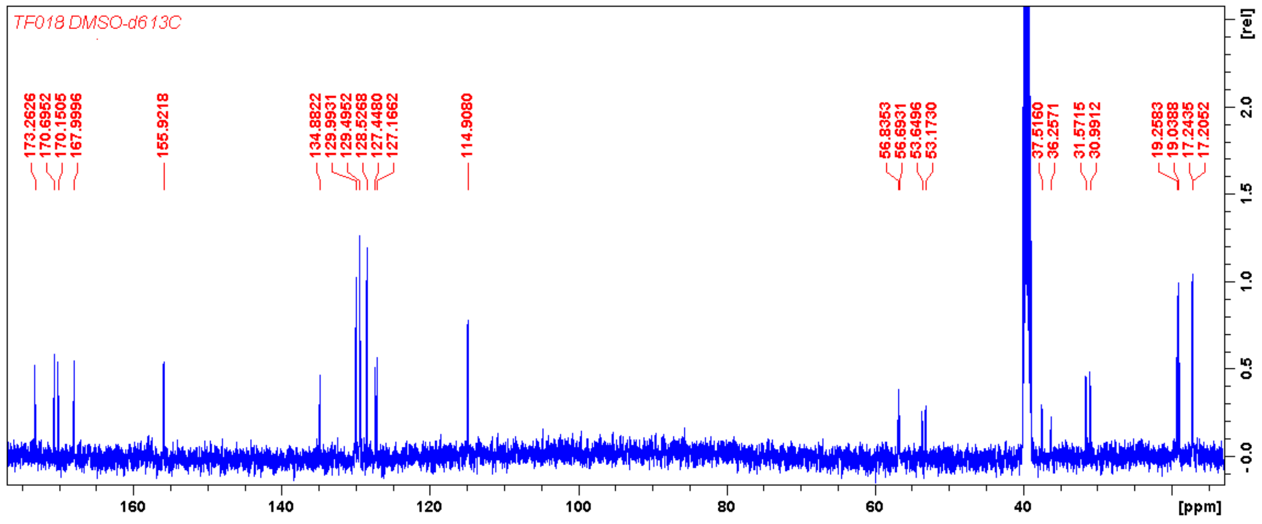

Figure S29. ^{13}C NMR spectrum of Tyr-Val-Val-Phe (**8**) (500.13 MHz, DMSO).

Table S2. ^{1}H and ^{13}C NMR data for **8** (500 MHz, DMSO) (δ in ppm, *J* in Hz).

8

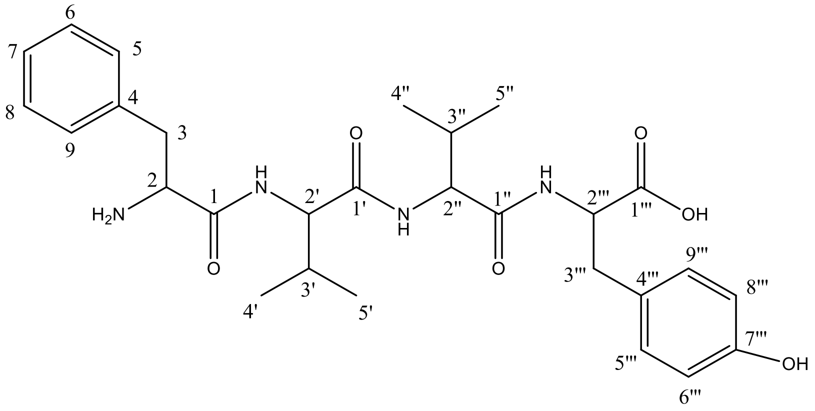

**Position**	**^{1}H δ (m, *J*)**	**^{13}C δ**
Phe		
1	-	160.0
2	4.24 (*t*, *J* = 7.6)	53.2
3	3.04 (*dd*, *J* = 14.0, 7.0)	37.5
	2.89 – 2.95 (*m*)	
4	-	134.9
5,9	7.23 – 7.33 (*m*)	129.5
6, 8	7.23 – 7.33 (*m*)	127.4
7	7.23 – 7.33 (*m*)	127.2
Val		
1’	-	170.7
2’	4.32 (*dd*, *J* = 9.4, 5.7)	56.8
3’	1.80 – 1.86 (*m*)	31.6
4’	0.67 (*d*, *J* = 7.0)	19.0
5’	0.52 (*d*, *J* = 6.8)	17.2
NH	8.32 (*d*, *J* = 8.3)	-
Val		
1”	-	170.1
2”	4.54 (*dd*, *J* = 9.4, 5.0)	56.7
3”	1.73 – 1.79 (*m*)	31.0
4”	0.66 (*d*, *J* = 6.8)	19.2
5”	0.63 (*d*, *J* = 6.9)	17.2
NH	8.45 (*d*, *J* = 9.1)	-
Tyr		
1”’	-	173.3
2”’	4.34 – 4.39 (*m*)	53.4
3”’	2.89 – 2.95 (*m*)	36.2
	2.66 (*dd*, *J* = 14.0, 9.7)	
4”’	-	128.5
5”’,9”’	6.99 (*d*, *J* = 8.5)	130.0
6”’, 8”’	6.61 (*d*, *J* = 9.1)	114.9
7”’	-	155.9
NH	8.10 (*d*, *J* = 9.2)	-

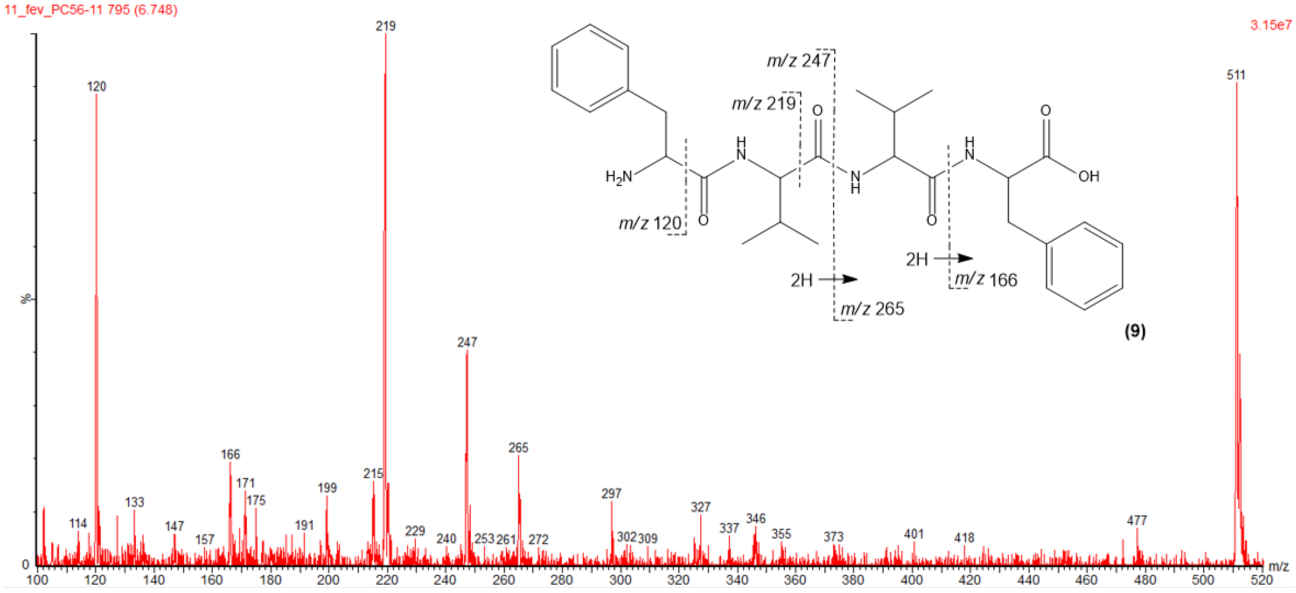

Figure S30. MS/MS spectrum of Phe-Val-Val-Phe (**9**).

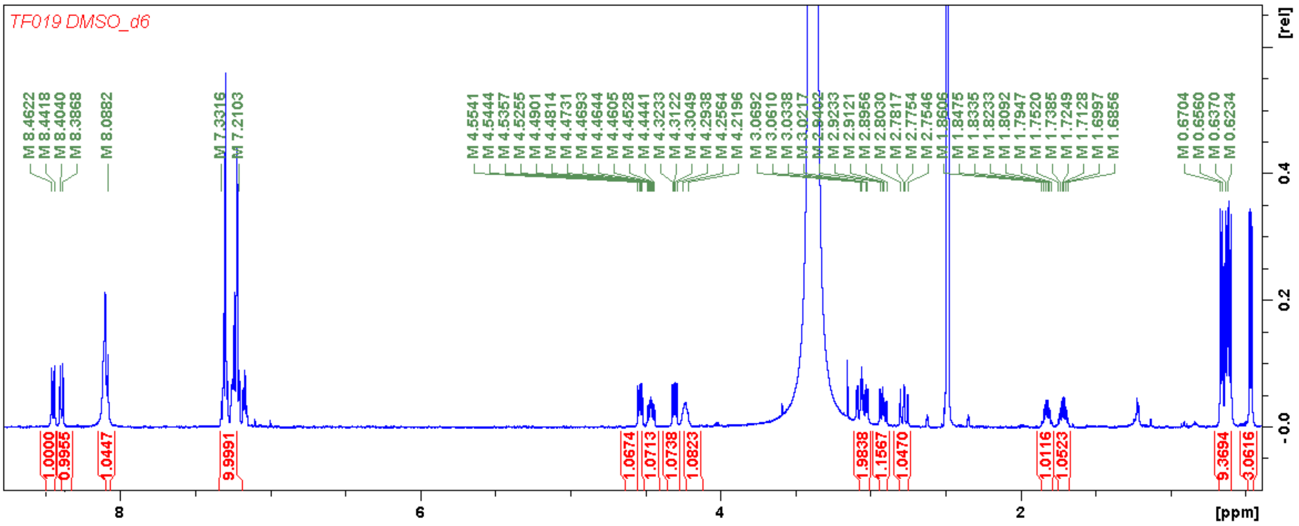

Figure S31. ^{1}H NMR spectrum of Phe-Val-Val-Phe (**9**) (500.13 MHz, DMSO).

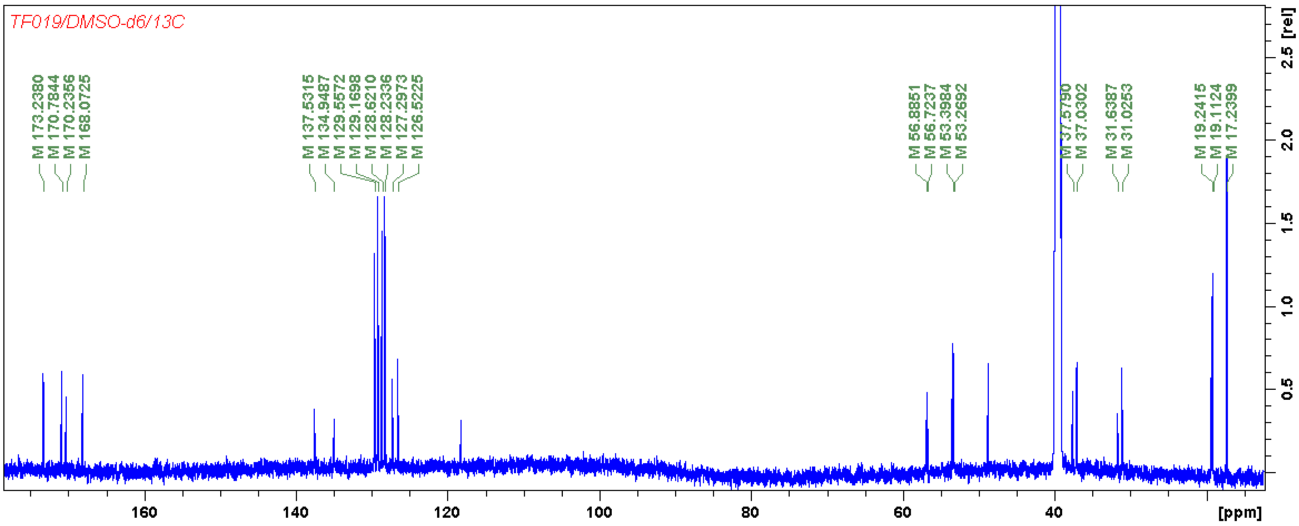

Figure S32. ^{13}C NMR spectrum of Phe-Val-Val-Phe (**9**) (500.13 MHz, DMSO).

Table S3. ^1H and ^{13}C NMR data for **9** (500 MHz, DMSO) (δ in ppm, *J* in Hz).

9

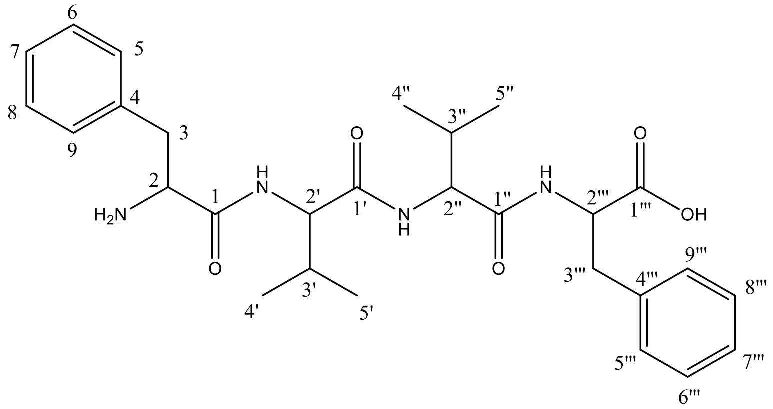

Position	^1H δ (m, *J*)	^{13}C δ
Phe		
1	-	168.1
2	4.21 – 4.25 (m)	53.3
3	3.04 (dd, J = 6.1, 13.7)	37.0
	2.77 (J = 10.7, 13.8)	
4	-	134.9
5,9	7.21 – 7.33 (m)	129.2
6, 8	7.21 – 7.33 (m)	128.6
7	7.21 – 7.33 (m)	127.3
Val		
1’	-	170.2
2’	4.31 (dd, J = 5.5, 9.2)	56.7
3’	1.71 (m)	31.0
4’	0.63 (d, J = 6.8)	19.1
5’	0.60 (d, J = 6.8)	19.2
NH	8.45 (d, *J* = 10.2)	-
Val		
1”	-	170.7
2”	4.54 (dd, J = 4.8, 9.2)	56.9
3”	1.82 (m)	31.6
4”	0.66 (d, J = 7.2)	17.3
5”	0.45 (d, J = 7.0)	17.3
NH	8.39 (d, *J* = 8.6)	-
Phe		
1”’	-	173.2
2”’	4.47 (ddd, J = 4.3, 8.5, 18.7)	53.4
3”’	3.07 (dd, J = 4.4, 14.1)	37.6
	2.91 (dd, J = 8.5, 14.0)	
4”’	-	137.5
5”’,9”’	7.21 – 7.33 (m)	129.5
6”’, 8”’	7.21 – 7.33 (m)	128.2
7”’	7.21 – 7.33 (m)	126.5
NH	8.01 (s)	-

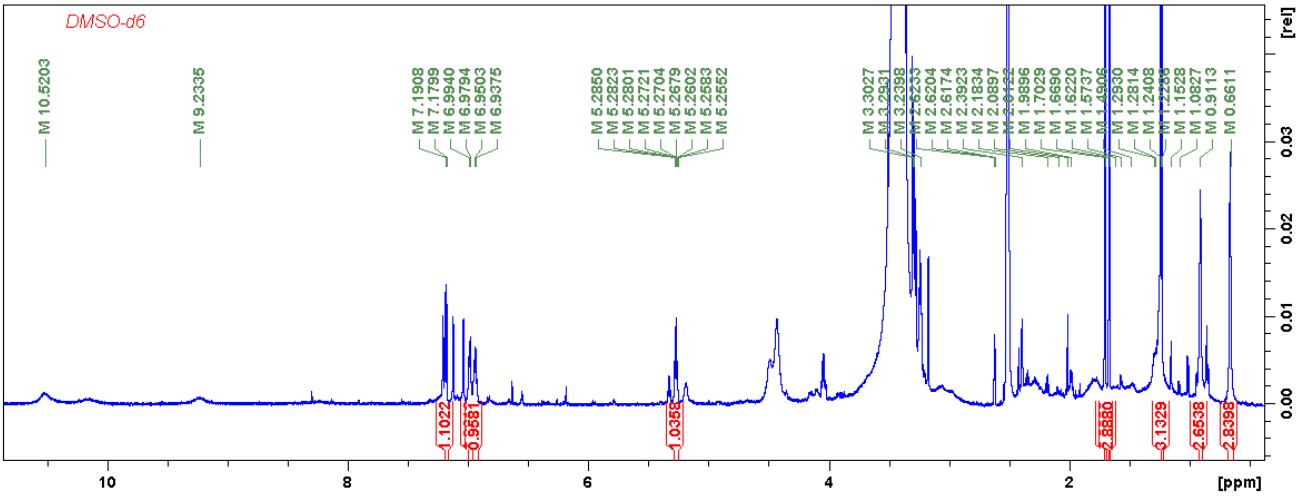

Figure S33. ^{1}H NMR spectrum of chrysogenamide A (**10**) (500.13 MHz, DMSO).

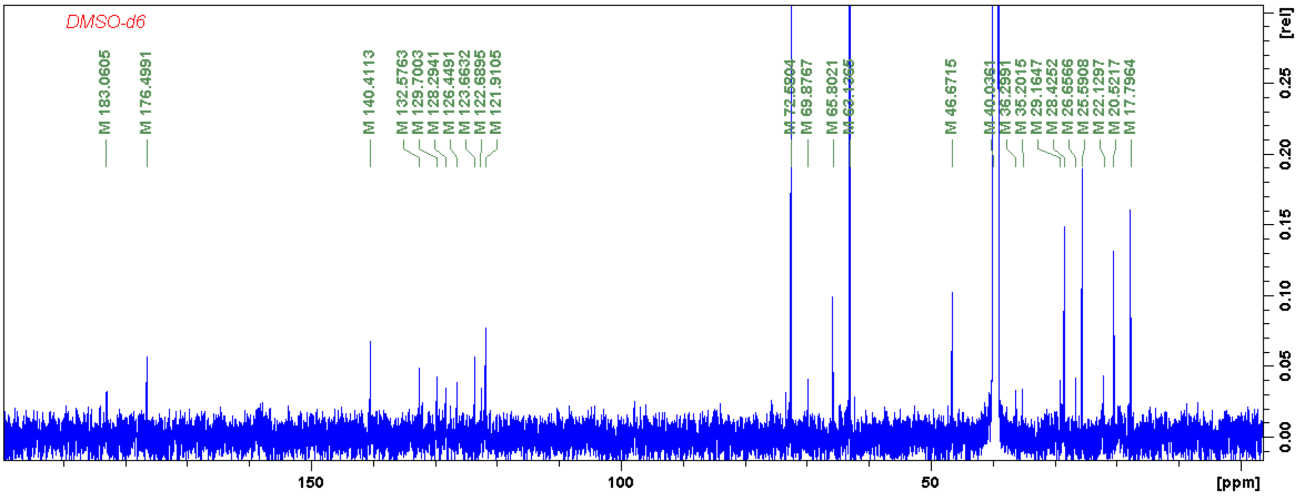

Figure S34. ^{13}C NMR spectrum of chrysogenamide A (**10**) (500.13 MHz, CDCl3).

Table S4. ^{1}H and ^{13}C NMR data for **10** (500 MHz, DMSO) (δ in ppm, *J* in Hz).

10

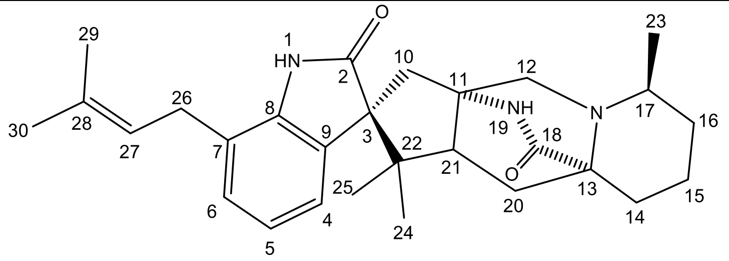

Position	^{1}H δ (m, *J*)	^{13}C δ
1-NH	10.52 (*s*)	-
2	-	183.1
3	-	72.6
4	7.19 (*d, J* = 7.2)	126.4
5	6.94 (*d, J* = 7.7)	121.9
6	6.98 (*d, J* = 7.4)	128.3
7	-	123.6
8	-	140.4
9	-	129.7
10		40.0
11	-	69.9
12a	3.30 (*d = 5.8*)	63.1
12b	2.39 (*m*)	-
13	-	65.8
14a	1.49 (*m*)	26.6
14b	1.15 (*m*)	-
15a	2.09 (*m*)	29.2
15b	10.52 (*s*)	-
16a	1.57 (*m*)	35.2
16b	1.08 (*m*)	-
17	1.99 (*m*)	65.8
18	-	176.5
19-NH	9.23 (*s*)	-
20a	1.62 (*m*)	36.3
20b	1.28 (*m*)	-
21	2.62 (*m*)	46.7
22	-	46.7
23	1.23 (*d, J* = 6.9)	22.1
24	0.66 (*s*)	20.5
25	0.91 (*s*)	20.5
26	3.24 (*m*)	28.4
27	5.27 (*tt, J* = 7.3, 1.3)	122.7
28	-	132.6
29	1.70 (*s*)	25.5
30	1.67 (*s*)	17.8

S4. Antifungal assays

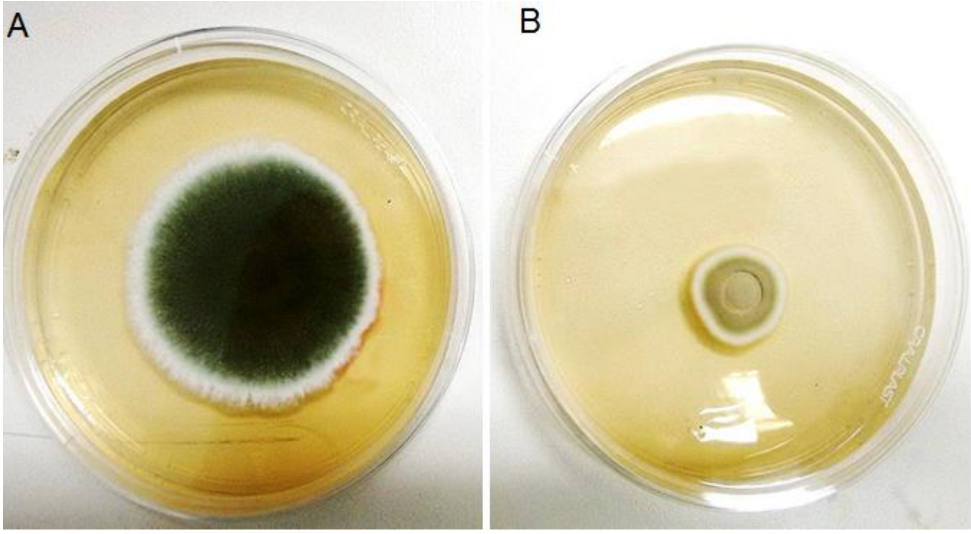

Figure S35. *P. digitatum* growing on (A) PDA and (B) PDA with 0.5 mg ml^{-1} of the co-cultive extract. *P. digitatum* exhibited reduction in radial growth of 67% in presence of *P. citrinum* metabolites

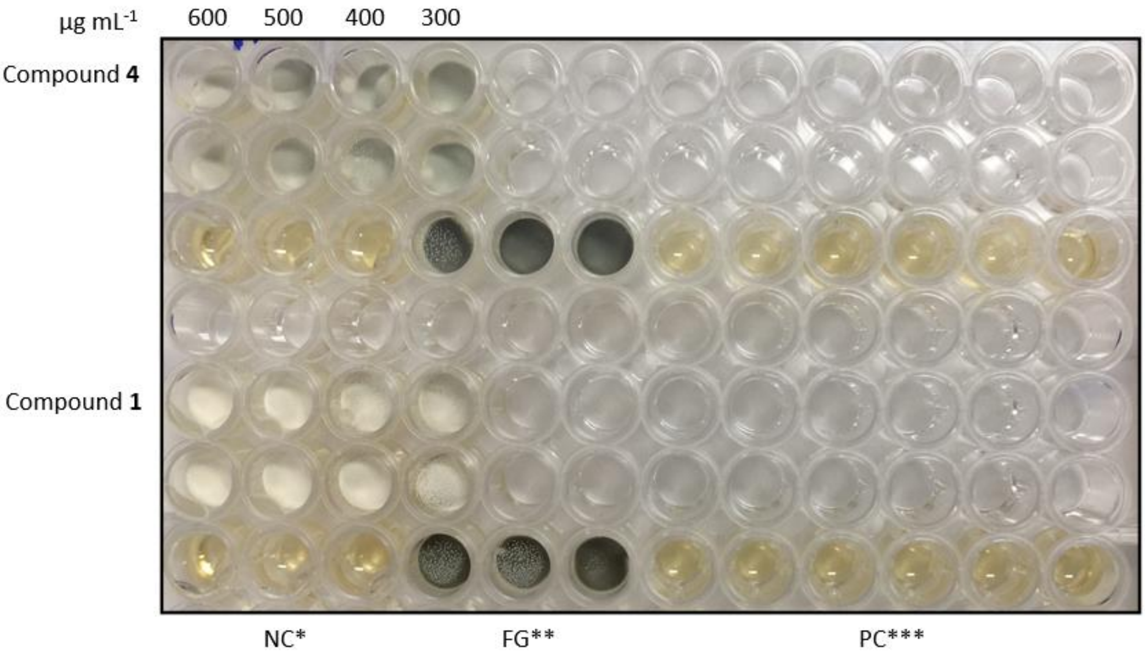

Figure S36. Microplate of MIC assay of tryptoquialanine A **(1)** and 15-dimethyl-2-epi-fumiquinazoline A **(4)** against *P. citrinum*. *NC is the negative control (no inoculation). **FG is the fungal control in YES media and 5% methanol. ***PC is the positive control (50 µg mL^{-1} of itraconazole).

S5. Confocal Laser Scanning Microscopy

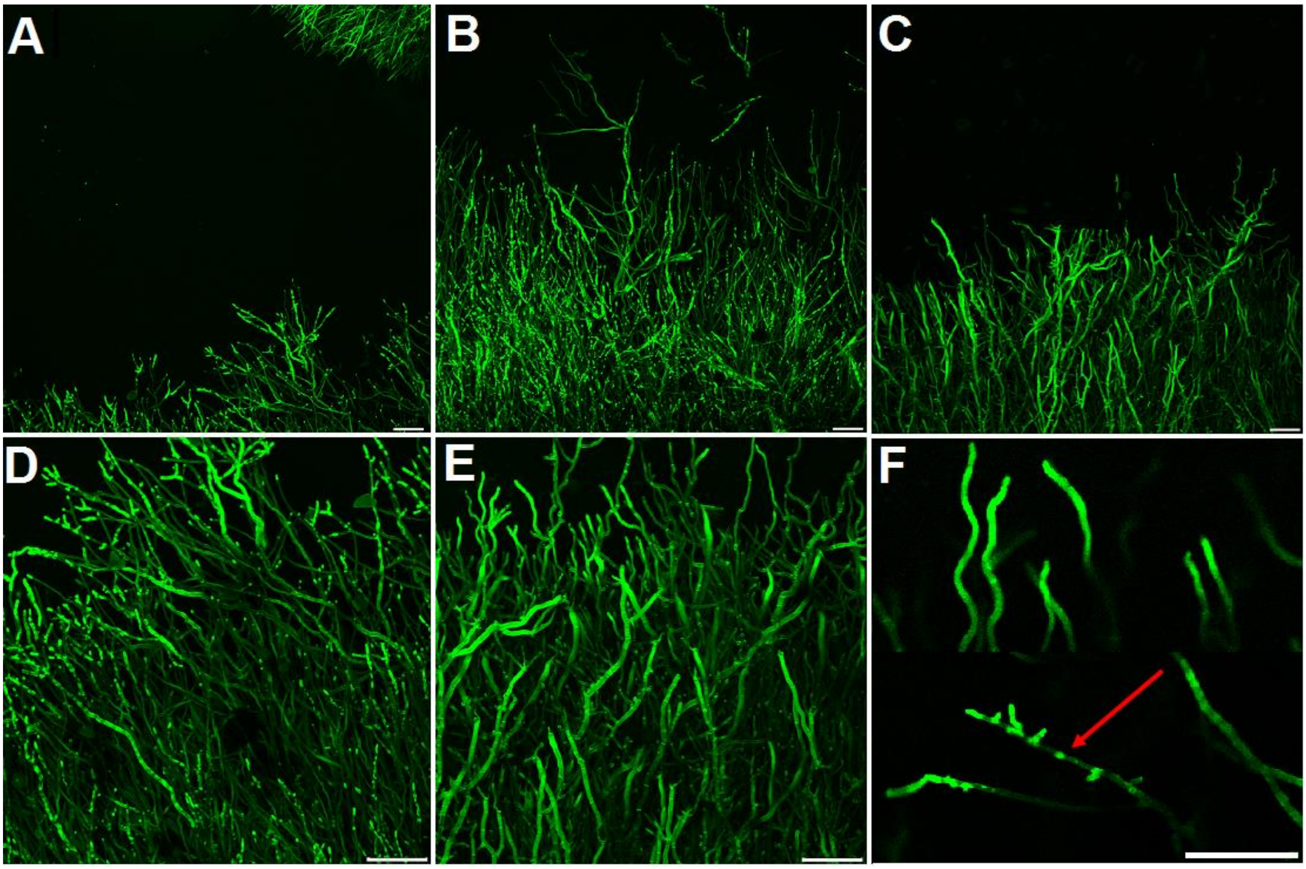

Figure S37. Confocal laser scanning microscopy of Congo Red-stained co-culture of *P. digitatum* and *P. citrinum*. (A) Interface zone between PD (below) and PC (above). PD hyphae: (B) sample and (C) control. Zoom in: (D) PD sample and (E) PD control. (F) Comparison between PD hyphae in control (above) and in sample (below): patches of Congo Red indicates a defective fungal cell wall. Bars = 5.0 µm
